# Beyond integration: modeling every pixel to obtain better structure factors from stills

**DOI:** 10.1107/S2052252520013007

**Published:** 2020-10-24

**Authors:** Derek Mendez, Robert Bolotovsky, Asmit Bhowmick, Aaron S. Brewster, Jan Kern, Junko Yano, James M. Holton, Nicholas K. Sauter

**Affiliations:** a Molecular Biophysics and Integrated Bioimaging Division (MBIB), Lawrence Berkeley National Laboratory, Berkeley, CA 94720, USA; b Stanford Synchrotron Radiation Lightsource, SLAC National Accelerator Laboratory, Menlo Park, CA 94025, USA; c Department of Biochemistry and Biophysics, UC San Francisco, San Francisco, CA 94158, USA

**Keywords:** serial crystallography, free-electron lasers, SFX, stills, data processing

## Abstract

Most crystallographic data processing methods use pixel integration. In serial femtosecond crystallography (SFX), the intricate interaction between the reciprocal lattice point and the Ewald sphere is integrated out by averaging symmetrically equivalent observations recorded across a large number (10^4^−10^6^) of exposures. Although sufficient for generating biological insights, this approach converges slowly, and using it to accurately measure anomalous differences has proved difficult. This report presents a novel approach for increasing the accuracy of structure factors obtained from SFX data. A physical model describing all observed pixels is defined to a degree of complexity such that it can decouple the various contributions to the pixel intensities. Model dependencies include lattice orientation, unit-cell dimensions, mosaic structure, incident photon spectra and structure factor amplitudes. Maximum likelihood estimation is used to optimize all model parameters. The application of prior knowledge that structure factor amplitudes are positive quantities is included in the form of a reparameterization. The method is tested using a synthesized SFX dataset of ytterbium(III) lysozyme, where each X-ray laser pulse energy is centered at 9034 eV. This energy is 100 eV above the Yb^3+^ *L*-III absorption edge, so the anomalous difference signal is stable at 10 electrons despite the inherent energy jitter of each femtosecond X-ray laser pulse. This work demonstrates that this approach allows the determination of anomalous structure factors with very high accuracy while requiring an order-of-magnitude fewer shots than conventional integration-based methods would require to achieve similar results.

## Abstract 2

A pixel-based approach for extracting structure factors from X-ray free-electron laser crystal diffraction measurements is presented.

## 1. Introduction   

The accuracy of structure factor estimation remains a central experimental focus as we approach the tenth anniversary of serial femtosecond X-ray crystallography (SFX) for biological structure determination at X-ray free-electron lasers (XFELs, Chapman *et al.*, 2011 ▸). Ultrafast X-ray pulses from such facilities provide unique opportunities to investigate functional, room-temperature protein states, while probing enzyme dynamics on time scales from femtoseconds to milliseconds (Alonso-Mori *et al.*, 2016 ▸; Pande *et al.*, 2016 ▸; Thomaston *et al.*, 2017 ▸; Stagno *et al.*, 2017 ▸; Tosha *et al.*, 2017 ▸; Nogly *et al.*, 2018 ▸; Kern *et al.*, 2018 ▸; Nango *et al.*, 2019 ▸; Dasgupta *et al.*, 2019 ▸; Ibrahim *et al.*, 2020 ▸), yet largely avoiding radiation damage (Chapman *et al.*, 2014 ▸; Spence, 2017 ▸; Fransson *et al.*, 2018 ▸). New protein science discoveries commonly arise at the extreme limit of what the signal-to-noise of the diffraction data can support, as illustrated by our recent experiences with photosystem II (PSII, Kern *et al.*, 2018 ▸; Ibrahim *et al.*, 2020 ▸). There, the electron density revealed small time-dependent changes, including the appearance of a single substrate oxygen atom at the catalytic site against the backdrop of a 23-polypeptide protein complex. Efforts to assign rigorous uncertainties in atomic positions (Ibrahim *et al.*, 2020 ▸) showed significant structural changes, yet a clear desire remained to utilize the weaker data at the limiting resolution in order to gain further atomic insights. Ultimately, our interest lies in single-electron transfers at the four Mn positions of the PSII catalytic cofactor. Spatially resolving the *K* absorption edge individually for each Mn center has the potential to elucidate the electronic environment of each Mn atom (Sauter *et al.*, 2020 ▸). Such challenging measurements would require quantifying structure factor intensities between Friedel pairs 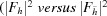) and among different states at the 1% level of uncertainty.

**Figure 1:**
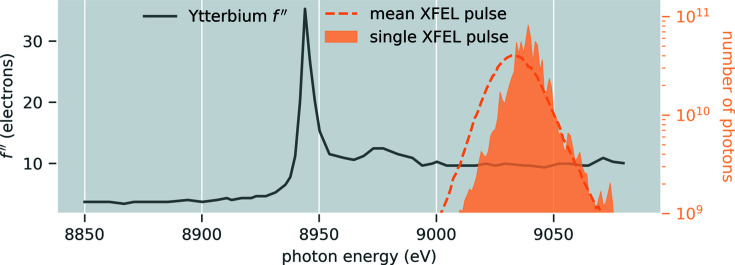
Anomalous dispersion curve for ytterbium and representative XFEL pulse spectra. Shown by the solid gray line is the measured ytterbium 3+ anomalous correction term [imaginary component; Shapiro *et al.* (1995 ▸)]. This curve was used in the data synthesis to compute the anomalous protein structure factors. For each synthesized diffraction pattern, we used a unique XFEL pulse, with the mean energy spectrum given by the dashed orange line. The spectrum of each XFEL pulse was randomly selected from a set of 100 000 real SASE spectra that were measured at the Linac Coherence Light Source (LCLS) and shifted to a high-energy remote for ytterbium *L*-III absorption (9034 eV). A single SASE spectrum is also shown for reference, illustrating the energy variation and intensity variation observed on a per-shot basis at the XFEL. At this energy the anomalous scattering is essentially constant within the range of the SASE energy variation. If the experiment had been done with the XFEL tuned to the absorption edge (8944 eV), then each diffraction pattern would have a varying level of anomalous scattering as per the *f*′′ curve.

Despite this experimental need for accurate interpretation of weak signals, a close look at data analysis pipelines has revealed stubborn and inherent difficulties specific to XFEL diffraction. In general terms, the structure factor amplitude is proportional to the square root of the Bragg spot intensity recorded for Miller index **h** = (*h*, *k*, *l*),
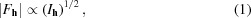
with the proportionality modulated by factors such as incident X-ray intensity, crystal size, polarization and absorbance (Darwin, 1922 ▸; Holton & Frankel, 2010 ▸). In particular, the interplay of incident beam divergence, X-ray spectrum and crystal mosaicity produces a distribution of diffracted intensities around regions in reciprocal space which satisfy Bragg’s condition. The success of the rotation method of data acquisition (Arndt & Wonacott, 1977 ▸), long practiced at synchrotron sources, rests on the ability to fully rotate the crystal through the angular range of this distribution, termed the ‘rocking curve’, while summing the diffracted intensity. In this way, the spectral shape and the crystal’s mosaic disorder do not contribute to the measurement error, as they are integrated out. Other factors, such as the intensity profile of the incident beam and the size and shape of the illuminated volume vary smoothly with the crystal rotation, hence scaling virtually eliminates errors due to these effects (Evans & Murshudov, 2013 ▸). In contrast, the lack of finite rotation during femtosecond exposure gives rise to partial observations that sample each Bragg spot’s rocking curve at a single position, meaning the sources of error described above all contribute to the uncertainty in SFX data. As expressed in the work by Kirian *et al.* (2010 ▸), for SFX, averaging repeated measurements of the same Miller indices across different diffraction patterns can minimize these uncertainties, assuming each measurement samples a rocking curve at random. However, when considering an SFX dataset in its entirety, duplicate measurements of a given Miller index form a skewed distribution peaking near zero, resulting from the many partial observations of the rocking curve tails that are low-photon count and contribute mostly noise, making it difficult to derive an ‘average’ intensity value that accurately represents |*F*_*h*_|^2^; see especially Figs. 1 and 5 in the work by Sharma *et al.* (2017 ▸) and Fig. 8 in the work by Kroon-Batenburg *et al.* (2015 ▸). Further contributing to noise, the rocking curves sampled in each shot are slight variations of one another, owing to the stochastic nature of XFEL pulses and the morphological variations amongst measured crystals.

Indeed, Kirian *et al.* (2010 ▸) acknowledges that the baseline technique of simply averaging duplicate measurements, the so-called ‘Monte Carlo’ approach, requires 10^4^–10^5^ individual diffraction patterns for the crystallographic *R* factor to converge; the 2010 paper thus ushered in ten years of technology development to identify improved algorithms. Most of these algorithms derive a physical model of the data, scored by one of three metrics: the ability to predict Bragg spots in the correct position, the self-consistency of equivalent Bragg spot intensities and, ultimately, the focus in our paper, the ability to predict not only the position of spots, but their size, shape and intensity profile.

Regarding the first metric, the ability to predict spots close to their observed positions requires knowledge of the unit-cell parameters and the crystal orientation in order to select those reciprocal lattice points in the diffracting condition (‘on the Ewald sphere’), and also to know the mosaic parameters (mosaic domain size and mosaic rotational spread) to determine which lattice points offset from the Ewald sphere can still generate reflections (Sauter *et al.*, 2014 ▸). Parameter refinement can optimize the positional match of data and model, which results in more accurate structure factors (Sauter *et al.*, 2014 ▸; Yefanov *et al.*, 2015 ▸; Waterman *et al.*, 2016 ▸; White *et al.*, 2016 ▸; Brewster *et al.*, 2018 ▸).

To examine the self-consistency of symmetrically equivalent Bragg spot intensities, efforts have been made to estimate the ‘partiality’ of each spot, *i.e.* a per-reflection scale factor that properly accounts for Ewald offset, where lattice points farther from ideal diffraction (and thus weaker) get multiplied by a larger factor to put duplicate observations on the ‘full spot’ scale. Several programs have emerged to treat the problem of partiality, with *cxi.merge* (Sauter, 2015 ▸), *prime* (Uervirojnangkoorn *et al.*, 2015 ▸) and *nXDS* (Kabsch, 2014 ▸) using the cell and mosaicity parameters already mentioned, paired with a monochromatic beam; whereas *ccpxfel* (Ginn *et al.*, 2016 ▸) and *partialator* (White, 2014 ▸) offer a polychromatic model intended to model the spectral width produced by the self-amplified spontaneous emission (SASE) obtained at XFEL sources (Emma *et al.*, 2004 ▸; Margaritondo & Ribic, 2011 ▸). All of these programs employ post-refinement, iteratively optimizing the parameters such that multiple partiality-corrected measurements of the same Bragg reflection yield the most consistent integrated intensity values after scaling.

Finally, the program *EVAL* exemplifies profile fitting (Duisenberg *et al.*, 2003 ▸; Schreurs *et al.*, 2010 ▸; Kroon-Batenburg *et al.*, 2015 ▸), utilizing a detailed physical description (source divergence, source dispersion, crystal size, mosaic block size and mosaic rotational spread) to faithfully model the size, shape and intensity profile of each Bragg spot. Along with other diffraction modeling programs such as *SIM_MX* (Diederichs, 2009 ▸), *pattern_sim* (White *et al.*, 2012 ▸) and *nanoBragg* (Holton *et al.*, 2014 ▸; Lyubimov *et al.*, 2016 ▸), *EVAL* allows us to explore how each of these physical parameters influences the appearance of Bragg spots (see also Nave, 1998 ▸, 2014 ▸), whereas the *EVAL* paper goes further and uses the profile-derived corrections in a simplex minimization to simultaneously optimize unit cells, crystal orientations and per-shot scale factors, achieving an optimal set of corrections for already-integrated spots.

Conventional data processing involves a two-step approach, first measuring Bragg spots on individual images, and then scaling and merging them together. For the work presented in this paper, determination of optimal structure factors |*F*_**h**_|, given the data, occurred in a single step, guided by a likelihood target function dependent on crystal orientations, unit cells, scale factors, mosaic parameters, photon spectra and, importantly, a starting list of structure factors provided by conventional data processing. The likelihood target received contributions from pixels across all images,^1^ with computation of per-pixel probabilities facilitated by the forward modeling program *nanoBragg*. A new program, *diffBragg*, provided the first derivatives of the forward model with respect to the various parameters, allowing effective navigation of the likelihood gradient in order to deduce the multi-parameter maximum likelihood. In using a likelihood formalism at the pixel level, we utilized an explicit error model for each pixel derived from first principles, hence optimization of the errors, which depend on the model, occurred at each refinement step. Also, the joint refinement of all parameters correctly accounted for covariance. For example, structure factor amplitudes and per-shot scale factors both directly increase the intensities of spots; however, in the global treatment, other shots measuring the same reflections constrain the structure factors. The integration methods described above all reduce the complicated spot profiles to single numbers; however, in the *diffBragg* approach, each pixel in the spot profile is tied directly to the model, significantly increasing the number of observations used during model optimization, and leading to higher parameter accuracy from fewer overall shots.

Pixel-level refinement can resolve extremely sensitive details, such as the dispersion corrections arising from two differently oxidized metal atoms, as shown with synthetic data in the work by Sauter *et al.* (2020 ▸). Having potential access to this level of detail from Bragg scattering opens up new and exciting experimental avenues to consider as the next decade of SFX science begins. Here, using the newly developed tool *diffBragg* on synthetic data, we further advanced the pixel refinement approach to extract more information from fewer recorded shots: we optimized a set of 13 704 structure factor amplitudes using 2023 XFEL diffraction patterns to a comparable accuracy to that achieved from conventional integration of 19 953 diffraction patterns, and we assumed no prior knowledge other than a positivity restraint applied to the structure factors. Furthermore, with the new protocol we combined positional refinement and post-refinement in a single framework. Sections 2.1 and 2.2 below describe the creation of synthetic data with *nanoBragg*; Section 2.3 introduces the new framework, *diffBragg*, for iterative parameter fitting. Section 3 details the improvement afforded by the new method, beyond the initial inputs from the program *dials.stills_process* [Brewster *et al.* (2016 ▸), see also Brewster, Young *et al.* (2019 ▸) for a recent description of the graphical user interface], which represent conventional data analysis. The results presented here required significant computational resources to achieve; however, GPU-acceleration, a near-term goal, will help in this regard (see Section 3.2.3). In addition to setting the ground work for using first-derivatives to perform more complicated refinements, this work reveals the potential to extract sensitive information from fewer recorded shots.

## 2. Methods   

### 2.1. Components of synthetic data   

To test the approach, we synthesized realistic lysozyme Yb^3+^ XFEL diffraction images with a mean XFEL pulse energy of 9034 eV, just above the Yb^3+^ *L*-III absorption edge (Fig. 1 ▸). We chose an anomalous dataset as a stringent test, as anomalous differences are highly sensitive to errors in structure factor estimation.

#### 2.1.1. Protein   

We used the program *CCTBX* [*Computational Crystallography Toolbox*, Grosse-Kunstleve *et al.* (2002 ▸)] to generate structure factors [see equation 1 of Sauter *et al.* (2013 ▸) for details] from PDB entry 4bs7, a room temperature lysozyme derivative structure with two Yb^3+^ sites (Pinker *et al.*, 2013 ▸). Wavelength-independent scattering factors for all atoms were calculated using Cromer–Mann coefficients [as tabulated in the work by Brown *et al.* (2006 ▸)], and anomalous scattering factor corrections *f*′ and *f*′′ for all protein atoms were computed using the Henke tables (Henke *et al.*, 1993 ▸). However, for ytterbium we specifically used *f*′ and *f*′′ from measured data (Shapiro *et al.*, 1995 ▸; Hendrickson & Ogata, 1997 ▸). Fig. 1 ▸ shows the experimentally determined Yb profile of *f*′′, with a magnitude at the high-energy remote (9034 eV) of 10 electrons (Fig. 1 ▸).

#### 2.1.2. Crystal   

We synthesized data from tetragonal lysozyme, with the unit cell *a* = *b* = 79.1, *c* = 38.4 Å. Each synthetic crystal had mosaic domains consisting of 1000 unit cells (10 along each crystal lattice axis). The crystal mosaic spread was computed by averaging scattering contributions from 100 equivalent mosaic domains. The misorientation of each mosaic domain with respect to the nominal orientation was taken from a normal distribution with a mean of 0° and standard deviation of 0.01° degrees, forming a mosaic texture. The mosaic texture was the same for all synthetic crystals, and the value 0.01° was assumed to be a typically observed value in real crystals (Bellamy *et al.*, 2000 ▸). For each shot, the total scattering from this mosaic average was multiplied by a random scale factor *Z* drawn from a normal distribution with mean μ_*Z*_ = 1150 and standard deviation σ_*Z*_ = 115 about the mean. This scale factor *Z* is the total number of mosaic domains in the crystal. The reason for using only 100 mosaic orientations for spread (instead of *Z*) was computational expediency. Random variation in the scale factor for each crystal was used to mimic variation in exposed crystal volume expected during each shot at the XFEL. The value 1150 was chosen by hand such that the contrast between low- and high-resolution spots was typical. We represented the crystal using a standard matrix convention (Busing & Levy, 1967 ▸), where each crystal orientation is represented by two matrices: an upper-triangular matrix **B**, whose columns specify the lattice basis vectors in an aligned orientation defined according to the convention of Arndt & Wonacott (1977 ▸), and a three-parameter rotation matrix **U**_*s*_ which moves the crystal from its aligned position into its observed position in shot *s*. For the tetragonal system
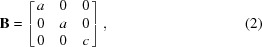
where *a* and *c* are the real-space unit-cell edge lengths.

#### 2.1.3. Background scattering   

We synthesized the diffraction akin to that measured under vacuum at the Coherent X-ray Imaging (CXI, Liang *et al.*, 2015 ▸) instrument at the Linac Coherent Light Source (LCLS), employing a gas dynamic virtual nozzle (GDVN) for sample delivery (DePonte *et al.*, 2008 ▸). The GDVN uses water pressure on a piston to force sample through a capillary and out of a nozzle to produce a liquid jet of sample in the interaction region. We assumed the jet was focused to a 5 µm diameter by a helium gas sheath. For background scattering we modeled the irradiated water volume as that of a cylinder: 5 µm in length multiplied by the beam spot size, a circle with a diameter of 1 µm. We neglected scattering from the helium sheath, and otherwise the path between the interaction region and detector was assumed to be a vacuum.

#### 2.1.4. Beam and detector   

We synthesized measurements from a 32-panel Cornell–Stanford Pixel Array Detector (CSPAD) as set up at CXI (Hart *et al.*, 2012 ▸), where each panel consists of 185 × 388 pixels, each 109.92 µm × 109.92 µm in size. We used a 2 × 2 pixel-oversampling rate to minimize aliasing errors from Bragg peaks whose signal might change significantly across the physical pixel dimension. We used a realistic three-dimensional CSPAD geometry obtained from CXI, such that the individual panels had relative rotations [see Brewster *et al.* (2018 ▸) for a detailed description]. We defined a ‘pixel measurement’ by both its position on the X-ray detector and the diffraction event it represents. Therefore, we let each pixel measurement be *X*_*i*,*s*_ where *s* refers to an X-ray event, or shot, and *i* is an index specifying the pixel position in the detector pixel array. The index *i* is equivalent to a triple index (*panel*, *fast*, *slow*) where *panel* is the CSPAD panel ID (0–31), *fast* is the panel fast-scan pixel coordinate (0–184) and *slow* is the panel slow-scan pixel coordinate (0–387). The CSPAD was placed 124 mm from the interaction region, giving a corner resolution of 1.7 Å; however, we only analyzed scattering out to 2.1 Å. For each synthesized diffraction event we used a unique SASE input spectrum that was a scaled and shifted version of real spectra recorded during an LCLS experiment (run 16, proposal number LS49) using an upstream spectrometer (Zhu *et al.*, 2012 ▸). Fig. 1 ▸ shows a representative XFEL pulse spectrum used to generate synthetic data, as well as the average spectrum. We assumed a uniform flux of photons with an average of 8 × 10^10^ photons per pulse, though the total fluence was different for each synthesized shot. We ignored beam divergence effects, as these are typically small at XFELs.

### 2.2. *nanoBragg*: computing synthetic data   

We used the program *nanoBragg* to compute, for each pixel during each shot, the expected Bragg scattering *I*_*i*,*s*,data_ and background scattering *T*_*i*,*s*,data_, and also to apply a realistic noise model.

#### 2.2.1. *nanoBragg*: generating Bragg scattering   

To compute the Bragg scattering measured by each pixel, *nanoBragg* applies the kinematic theory where the expected number of Bragg-scattered photons in each pixel is the product of the incident X-ray fluence with the scattering cross section of the crystal and the solid angle ΔΩ_*i*_ of the pixel. In what follows, *r*_e_ is the classical electron radius, κ_*i*_ is the Kahn polarization factor for scattered light from a pre-polarized incident source (Azároff, 1955 ▸; Kahn *et al.*, 1982 ▸), *J_s_*(λ) is the fluence in photons per area at wavelength λ, *Z_s_* is the crystal scale factor randomly sampled for each shot from a normal distribution 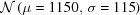, |*F*_**h**_| is the structure factor amplitude of the protein in each unit cell and *I*_0_(Δ**h**_*i*,*j*,*s*_, *m*) is the interference factor arising from the periodicity in the crystal lattice. The term Δ**h**_*i*,*j*,*s*_ is the Ewald offset at the pixel determined from the orientation of mosaic block *j*, and *m* ^3^ is the total number of unit cells in the mosaic domain. *N_j_* is the number of orientations used to form a mosaic texture, the scattering power of which is then normalized by *N_j_* and scaled up by *Z_s_* for total scattering equivalent to a crystal that is made up of *Z_s_* mosaic blocks. For the synthetic data used here, the expected Bragg scattering was computed according to
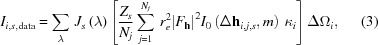
where the expression inside the square brackets is the scattering cross section of the whole crystal for photons of wavelength λ. The interference term *I*_0_(Δ**h**_*i*,*j*,*s*_, *m*) should be maximal when the pixel is exactly probing the diffraction condition (Δ**h**_*i*,*j*,*s*_ = 0), and it should fall off rapidly as Δ**h**_*i*,*j*,*s*_ increases. We let *I*_0_(Δ**h**_*i*,*j*,*s*_, *m*) take the Gaussian form
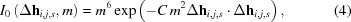
where the constant *C* was chosen such that the full width at half-maximum of the principal peaks in *I*_0_ would be equal to that arising from a parallelepiped mosaic block [see Appendix *A* [App appa] for a derivation of equation (4); in the work here, we used *C* = 3.175]. The dimensionless Ewald offset Δ**h**_*i*,*j*,*s*_ is simply the residual between the fractional Miller index at each pixel with the nearest integer Miller index
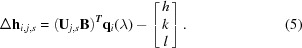
Here **q**_*i*_(λ) is the momentum transfer from incident beam unit vector 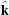 to the scattered beam unit vector 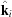 (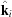 points from the interaction region to the pixel):
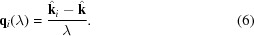
The transpose of **UB** in equation (5) is necessary as the columns of **B** are what define the lattice translation vectors. Note the dependence of *I*_0_(Δ**h**_*i*,*j*,*s*_, *m*) on *m*; a larger mosaic domain parameter yields a brighter maximum with a more rapid falloff. For the synthetic data, we let *m* = 10 and *N_j_* = 100. For real data, we expect the true shape of the Bragg peak profiles to be well approximated by Gaussians, or a sum of Gaussians. The parameters describing the synthetic Bragg scattering data are summarized in Table 1 ▸.

#### 2.2.2. *nanoBragg*: generating background scattering   

Background scattering was synthesized by estimating the total number of background molecules in the bulk background-scattering volume. Here, *nanoBragg* computed the expected scattering for liquid water:
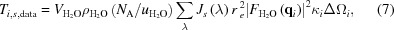
where *V*_H___2___O_, ρ_H___2___O_ and *u*_H___2___O_ are the volume, density and molecular weight of water, respectively, *N*_A_ is Avogadro’s constant, and |*F*_H___2___O_(**q** *_i_*)| is the isotropic structure factor amplitude per molecule of liquid water which depends on the scattering angle of the pixel, 2θ_*i*_ = sin^−1^(**q**_*i*_λ/2). |*F*_H___2___O_(**q** *_i_*)| was measured independently at the Advanced Light Source beamline 8.3.1 (MacDowell *et al.*, 2004 ▸) and adjusted to match the absolute calibration in the work by Clark *et al.* (2010 ▸).

#### 2.2.3. *nanoBragg*: generating realistic measurement noise   

With *nanoBragg*, we applied three types of measurement noise arising from the inherent randomness of photon counting, signal amplification and detector readout. These noise operations are computed sequentially, modeling the various stages of photon measurement. In the first measurement stage, shot noise produces counting error; the number of photons arriving in the pixel is a random number sampled from a Poisson distribution with mean *I*_*i*,*s*,data_ + *T*_*i*,*s*,data_. In the second stage, the charge produced by detected photons is amplified by each pixel slightly differently. The amplifiers are fixed in the circuitry, however there is always error in their calibration (gain). Here, the photon gain for each pixel was assigned by randomly selecting numbers from a normal distribution with a mean of 28 ADUs per photon and standard deviation equivalent to 3% of the mean, or 0.84 ADUs. These per-pixel gain values are typical for the CSPAD, and once selected they are fixed for all shots. Note that this type of calibration error assumes all pixels are the same physical size; pixel-to-pixel non-uniformity could be computed by adjusting the expected number of photons before computing the Poisson deviates above. Lastly, there is noise associated with detector readout due to electronic switching during the readout event itself and dark-charge accumulation during the exposure. CSPADs are dominated by dark current noise, but here we make no distinction and lump all errors associated with readout into a single Gaussian process. We included readout noise of the detector by adding a random number to the final computed values, where that random number was drawn from a normal distribution with a mean of 0 and a standard deviation of 3 ADUs. We modeled dark-subtracted data, where an average dark signal had already been subtracted from each shot, hence why the readout error fluctuates about 0. Per-pixel readout and counting noise terms fluctuate across every pixel and every shot; however, pixel gain calibration errors, though different for each pixel, are constant for all shots. It is typical to divide the observed pixel values by a nominal gain value for the entire camera (28 in this case) so that a unit pixel increment is the same size as the signal from a single photon, hence we have for the pixel value
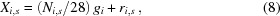
where the photon count *N*_*i*,*s*_ is randomly sampled from a Poisson distribution with the expectation value *I*_*i*,*s*,data_ + T_*i*,*s*,data_, the pixel gains *g_i_* form a normal distribution 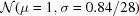, and the readout noise *r*_*i*,*s*_ is randomly sampled from a normal distribution 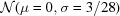. For a full description of the noise options available in *nanoBragg*, see the supplemental material from the work by Holton *et al.* (2014 ▸).

### 2.3. Maximum-likelihood structure factor estimation using pixel data   

**Figure 2:**
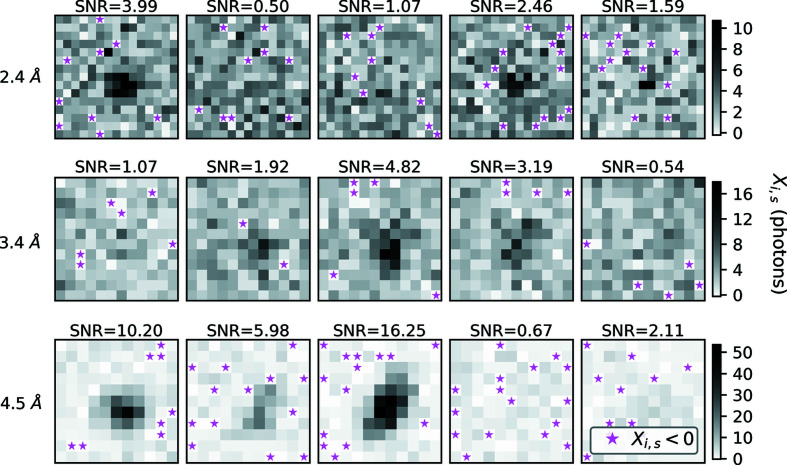
Bragg spot profile variation observed in synthetic XFEL images. Each row contains five shoeboxes centered on the same Miller index, yet measured on separate shots (*i.e.* from different crystals). The resolution of each Miller index is shown in the far left of each row. In standard data analysis, integrated signals from shoeboxes with equivalent Miller indices are averaged together, leading to large uncertainties in the resulting structure factor estimates. Signal-to-noise ratio (SNR) estimates for the Bragg reflections inside each shoebox are provided for reference. We computed SNR following the work by Leslie (1999 ▸) (using a weighted least-squares treatment to propagate the tilt-plane error). Pixels with negative readings are marked with a star. The background scattering was very low in the synthetic data, as we attempted to accurately replicate in-vacuum diffraction from crystals in a 5 µm liquid jet produced by a GDVN (DePonte *et al.*, 2008 ▸). Such low background in combination with per-shot readout errors gave rise to the negative pixels shown here. The gray scale represents *X*_*i*,*s*_ (observed pixel intensity in units of photons) as defined in equation (8). All sub-images in a row are on the same scale (indicated by the scale bars on the right in units of photons). Note, the middle row appears to have fewer negative readings, which we expect because it is on the water scattering ring and, as a result, those pixels receive more background scattering. Fig. S1 shows the same set of images before random noise was applied.

Fig. 2 ▸ shows several examples of the Bragg reflections from the synthetic diffraction patterns, illustrating the variability of repeated Bragg reflection measurements performed at XFELs (see Fig. S1 of the supporting information for the same images in the absence of noise). This per-shot variation in observations is what makes the analysis of XFEL still shots with conventional protocols subject to large uncertainties. By summing together neighboring Bragg spot pixels, one loses the intricate per-pixel intensity variations that encode the incident photon spectra and crystal morphology. This information could otherwise be used to constrain complicated physical models. Rather than integrate Bragg spots we seek to use their pixelated profiles to optimize a model, thereby disentangling the various contributions to the scattering, and obtaining a more accurate measure of the structure factor amplitudes |*F*_**h**_|.

#### 2.3.1. Maximum-likelihood target   

The goal was to treat our synthetic images as an experimental dataset, and then use the pixel values *X*_*i*,*s*_ within all the Bragg-spot shoeboxes (Fig. 2 ▸) to optimize a global model to obtain increased structure factor accuracy. We accomplished this through iterative parameter estimation, using a maximum-likelihood target. Let *p*(*X*_*i*,*s*_|Θ) represent the probability of observing *X*_*i*,*s*_ given the full set of model parameters Θ needed to describe the observed pixel values. We will explicitly define Θ in Section 2.3.7. The likelihood of the entire dataset is given by the product of the individual pixel probabilities
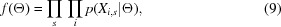
provided we neglect inter-pixel effects such as crosstalk: a fair approximation for the sake of defining an optimization target. Other inter-pixel effects such as point-spread are discussed in Section 4, but are not necessarily applicable. The set of parameters Θ_ML_ that maximizes the likelihood of the data 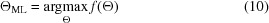
is called the maximum-likelihood estimate, or the most probable model, given the data.

#### 2.3.2. Probability of observing pixel values   

In order to express the likelihood of the data given by equation (9), we must define *p*(*X*_*i*,*s*_|Θ), the probability of observing *X*_*i*,*s*_ given a set of model parameters Θ describing the scattering. In this case we know the precise model that was used to generate *X*_*i*,*s*_, but the arguments below are general and applicable to real data. In what follows, we let *R_X_* represent a random variable for a pixel measurement, where *X*_*i*,*s*_ is a sample from the distribution governing *R_X_*. We assume *R_X_* describes an observation after division by the nominal gain, and after subtraction of an average dark pedestal, as shown in equation (8). Using the algebra of random variables, we can define *R_X_* as an expression involving three independent random variables:
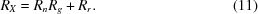
Here *R_n_* represents randomness in photon counting, *R_g_* represents randomness in signal amplification and *R_r_* represents randomness in signal readout. The random variable *R_n_* is governed by a Poisson distribution 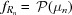 whose mean μ_*n*_ represents the expectation value for the number of photons captured by the pixel during the diffraction event. We can simplify the statistics by approximating 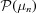 as a normal distribution with equivalent mean and variance: 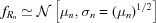. This approximation breaks down when the number of scattered photons approaches 0; however, in this regime we expect the readout term to dominate *R_X_*. The random variable *R_g_* arises due to error in detector gain calibration; even though we divide through by the nominal gain value, we do not know the precise gain of each pixel. Therefore, we let 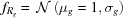 be the distribution governing the gain calibration error. One can estimate σ_*g*_ by recording flat illumination on the detector, *e.g.* scattering from a copper foil far upstream of the detector, but the result is never perfect. The product of the two normally distributed random variables *R_n_* *R_g_* is in general not a normal random variable, however in certain limits we can make that approximation. Specifically, if we assume that *R_n_* and *R_g_* are independent random variables, then for large μ_*n*_/σ_*n*_ and μ_*g*_/σ_*g*_ we can approximate (Seijas-Macías & Oliveira, 2012 ▸)
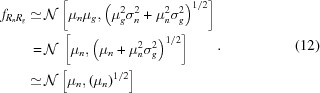
Note, we used the fact that μ_g_ = 1 and 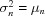 as shown above. The second approximation in equation (12), stating that 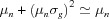 is valid for small σ_*g*_, yet becomes worse as μ_*n*_ increases. If 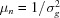, the approximation error is equivalent to μ_*n*_. For σ_*g*_ = 0.03 (0.84/28, which we used for the synthetic data) this occurs when μ_*n*_ = 1.1 × 10 ^3^ photons, and for the work presented here most pixels received far fewer photons. The random variable *R_r_* describes a random offset applied to each pixel measurement which is governed by the underlying electronics of the detector modules, and it follows a normal distribution 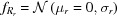. Usually during an experiment with a CSPAD, a dark measurement is recorded and subtracted from subsequent measurements. For a given exposure time this dark offset is generally stable, but there is always a random component that fluctuates on a shot-to-shot basis. This readout noise will result in a positive or negative offset applied to each pixel, and it is these offsets that are represented by *R_r_*. The value σ_*r*_ is easy to estimate by closing the X-ray shutter and observing the pixel values fluctuating in the absence of X-rays. We used a value of σ_*r*_ = 3/28 throughout the analysis in this paper, in line with equation (8). With the above definitions we now define the distribution *f*_*R*___X__ governing *R_X_*. This distribution is the convolution of 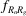 and *f*_*R_r_*_ (true for the sum of any two random variables). In the case that both *f_R_n___R_g__* and *f*_*R_r_*_ are normal, then *f*_*R_X_*_ will also be normal: 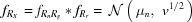, where 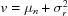 and * is a convolution operator. With this we can express the probability of observing *X*_*i*,*s*_ photons as
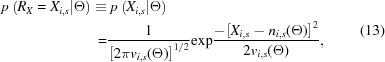
where we used the definition 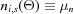 to be index-explicit, and where the model-dependent variance is given by
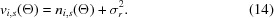
For the remainder of this report, we use *n*_*i*,*s*_(Θ) to represent the model for the expected number of photons in pixel *i* during shot *s*. Note this is different from *N*_*i*,*s*_ used in equation (8), which is a randomly drawn sample, given an expectation value *n*_*i*,*s*_(Θ). It is noteworthy that the probability model in equation (13) allows the observed data *X*_*i*,*s*_ to be negative, something mathematically forbidden when modeling the observations using Poisson statistics alone. In other words, a photon count by itself can never be negative, only when coupled with additional terms such as the readout noise can a pixel report a negative value. Negative pixel values occur in regions of weak scattering where readout noise dominates. This can easily happen and is indeed expected after dark current subtraction for in-vacuum low-background measurements at facilities like CXI (see Fig. 2 ▸).

#### 2.3.3. Selecting pixels for maximum-likelihood estimation   

In principle one can evaluate the likelihood shown in equation (9) for every pixel in the camera, but for the work shown here we only included a selection of pixels that were expected to be in the vicinity of Bragg scattering, referred to throughout this text as shoeboxes. Fig. 2 ▸ shows several such shoeboxes. Each synthetic CSPAD diffraction pattern is made up of 32 × 185 × 388 = 2 296 960 pixels, but by limiting the analysis to shoeboxes, we only used an average of 1.35 × 10^5^ pixels per image. This made maximum-likelihood estimation approximately 17 times faster than it would be if including all pixels. Interestingly, we found that over-predicting shoeboxes (in order to guarantee inclusion of all observed spots) did not hurt the refinement. This is a key distinction from integration methods, where over-prediction can be problematic.

#### 2.3.4. Modeling expected photons in each pixel   

We modeled *n*_*i*,*s*_(Θ) as a sum of Bragg scattering *I*_*i*,*s*_ and background scattering *T*_*i*,*s*_:
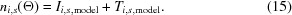
We will proceed to define the background and Bragg scattering models, after which we will define the full list of refinement parameters (summarized in Table 2 ▸). Note the subscript ‘model’ is used to distinguish these expressions from those in equations (3) and (7) describing the synthetic data.

#### 2.3.5. Bragg scattering model   

We modeled the Bragg scattering similarly to that shown in equation (3):[Disp-formula fd3]

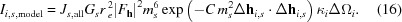
Here 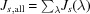 is the total fluence across all photon energies in the XFEL pulse, and 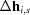 = 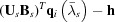 is computed using the central wavelength 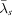 of each XFEL pulse and a single mosaic domain at orientation **U**_*s*_. The scale factor *G_s_* relates primarily to the crystal size variation, but other factors can also affect the scale during real measurement. One can use equation (4) directly to model the full energy spectra; however, here we use equation (16) purely for computational efficiency and accept that it is slightly inaccurate (see Section S1 and Fig. S2 of the supporting information). Also, given the relatively small mosaic domain size used for the synthetic data, we assumed mosaicity would dominate the spot profile shapes, as opposed to wavelength dispersion. Even though all crystals have the same mosaic parameter *m* and unit-cell matrix **B**, we modeled each crystal as having a unique mosaic parameter *m_s_* and unit-cell matrix **B**_*s*_.

#### 2.3.6. Background scattering model: tilt planes   

The measured background scattering arose from the solvent, and we did not model it using first principles. Instead, we fit a linear expression, or tilt plane, to the pixel measurements at the periphery of each Bragg spot shoebox (Rossmann, 1979 ▸), and the resulting tilt plane was used to evaluate the background intensity under the Bragg peaks. For fitting, we used weighted linear least squares. To obtain each tilt plane we solved the linear system
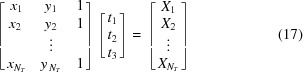
for the tilt plane coefficients **t** = [*t*_1_, *t*_2_, *t*_3_], where *x_i_*, *y_i_* are the fast-scan, slow-scan coordinates of the *N_T_* shoebox pixels selected for the tilt plane fit, and where *X_i_* are the observed values of those pixels. Rewriting equation (17) as **At** = **b**, we can then write the solution as 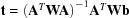 where **W** is a diagonal matrix whose diagonal entries are the reciprocals of crude variance estimates for each pixel value in **b**. Given a pixel value *X_i_*, we approximated its variance (for tilt plane fitting purposes only) as 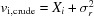, where σ_*r*_ is the readout noise standard deviation (3/28 = 0.11 in the synthetic data), and we used this information to compute a signal-to-noise estimate for each Bragg reflection (see Leslie, 1999 ▸). Fig. 2 ▸ shows these signal-to-noise estimates for several simulated reflections. Recalling equation (14), it becomes obvious that the estimate *v*_i,crude_ uses the approximation *n_i_* ≃ *X_i_*. In other words, *v*_i,crude_ approximates an expectation value with a single measurement, providing a suitable guess of the spot variance in the absence of a model. We emphasize that a unique background vector **t** was computed for each shoebox, that is, for each Bragg spot prediction on each shot. Ideally the pixels used in equation (17) do not include contributions from Bragg scattering. After solving for **t**, we modeled the background for pixels in the corresponding shoebox as
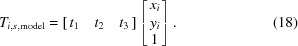
This linear fit exhibits no curvature and is therefore best applicable to local regions on the detector where the background signal is slowly varying. During the maximum-likelihood parameter optimization, the tilt plane coefficients [*t*_1_, *t*_2_, *t*_3_] were initialized as the solutions to equation (17) and were then fixed. Though the least squares solution is analytical, it is dependent on the proper distinction between background pixels and Bragg pixels. For weakly scattering data, tilt planes can be evaluated at levels close to zero intensity, sometimes giving rise to negative *n*_*i*,*s*_(Θ), especially for pixels at the shoebox periphery. This is a result of using a non-physical background model, and it poses a risk to violate equation (13) which requires that *v*_*i*,*s*_(Θ) remain positive. To guard against this occurrence, we filtered all shoeboxes whose tilt planes dipped below 0 for any pixel in the shoebox. This resulted in the removal of an average of 2.3 out of 580 shoeboxes per shot.

#### 2.3.7. Unknown model parameters   

We now explicitly define Θ, the list of all unknown model parameters that we determined via maximum likelihood. A parameter can be placed into one of two categories: local or global. A local parameter belongs to a particular XFEL diffraction shot. We let Γ_*s*_ represent the local parameters of shot *s*, namely the crystal orientation matrix **U**_*s*_, the unit cell matrix **B**_*s*_, the scale factor *G_s_* and the mosaic domain parameter *m_s_*:
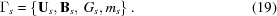
In total each Γ_*s*_ represents seven parameters: three Euler angles describing crystal orientation, two unknown unit-cell constants, a single scale factor and a single mosaic parameter. On the other hand, a global parameter is shared across all shots in the experiment. Global parameters in the model were the set of all structure factors, which we refer to here as {|*F*_**h**_|}. The full list of parameters for *N_s_* diffraction patterns was
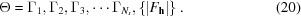
For the structure factor refinement, we imposed tetragonal symmetry, leading to 13 704 unique structure factor amplitudes out to 2.1 Å.

#### 2.3.8. Optimization using *diffBragg*   

Solving for Θ_ML_ given by equation (10) was carried out using the quasi-Newton optimization algorithm L-BFGS (Liu & Nocedal, 1989 ▸) as implemented in *CCTBX*. L-BFGS requires the first derivative of the likelihood expression *f*(Θ) in equation (9) with respect to each parameter of interest defined in equation (20). In practice it is typical to minimize the negative logarithm of the likelihood, both to maintain numerical accuracy when multiplying the large number of probabilities, and to make use of standard minimization algorithms. Therefore, we solved the equation
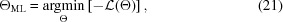
where
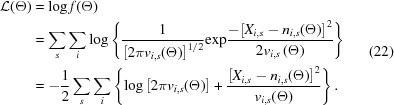
The first derivative of the log-likelihood for a parameter 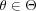 (needed for L-BFGS) is then given by [recalling equation (14)]
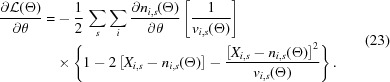
We developed a new program alongside *nanoBragg* (dubbed *diffBragg*) for computing the derivatives of the expected scattering *n*_*i*,*s*_ with respect to each parameter. For example, when computing the derivative of the likelihood expression with respect to the structure factors, we used *diffBragg* to evaluate
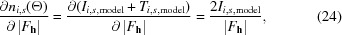
which follows from equation (16). The results were then substituted into equation (23) to compute the gradients of the likelihood expression needed for optimization.

It is important during refinement that the target function be equally sensitive to all parameters. To this end we applied reparameterizations of the form
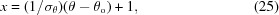
where θ_o_ is the initial value of the parameter and σ_θ_ represents the expected variation of the parameter during refinement (Hammersley, 2009 ▸). With this change of variables, all parameters started with an initial value of 1. If the target equation appeared exceptionally sensitive to certain parameters, the corresponding σ_θ_ values were incremented by factors of 10 until we observed the first several L-BFGS iterations updating parameters by sensible amounts. In this specific problem we also applied bound restraints to certain parameters. This was accomplished by reparameterizations of the form
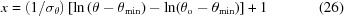
such that the parameter θ will always be greater than θ_min_ ≥ 0. For example, to ensure the structure factor amplitudes remained positive during refinement, we made the substitution 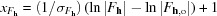. The parameters 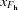 refined without restraints; however, the resulting 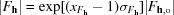 remained positive quantities. A similar restraint was used on the scales *G_s_* and mosaic parameters *m_s_*. With these reparameterizations the updated derivatives of the target equation can be written as
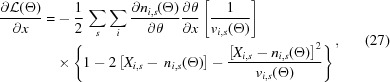
where ∂θ/∂*x* is computed for each parameter according to the corresponding reparameterization scheme. See Appendix *B* [App appb] for derivative expressions of the remaining model parameters. Optimization was carried out using the National Energy Research Scientific Computing Center (NERSC).

## 3. Results   

### 3.1. Initialization of model parameters   

**Figure 3:**
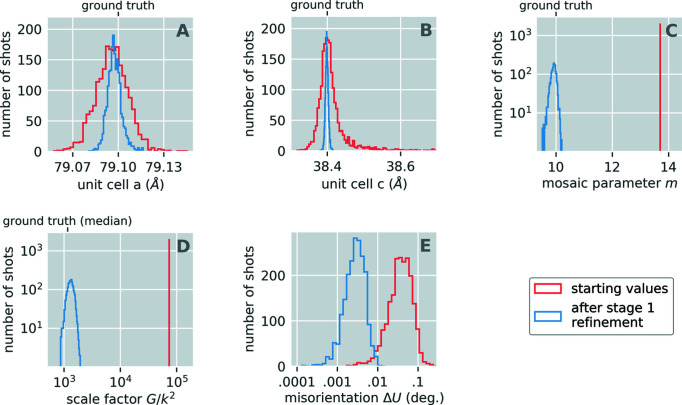
Stage 1 refinement results for 2023 exposures. During stage 1 refinement images are refined separately, resulting in a unique scale factor *G_s_*, mosaic domain parameter *m_s_*, unit cell matrix **B** *_s_* and rotation matrix **U** *_s_* for each crystal. Starting values are shown in red, and optimized values after stage 1 refinement are shown in blue. (*a*) and (*b*) Unit cell edges *a* and *c*. Initial values come from the *DIALS* indexing program (Winter *et al.*, 2018 ▸). Despite all crystals having the same unit cell (*a* = 79.1, *c* = 38.4 Å), the indexing results returned a distribution of cells. The unit cell *a*-edge refined to a median value of 79.097 Å, which is 0.003 Å different from the ground truth value. By modeling each shot with its known photon energy spectra, we indeed recovered a value of 79.100 Å for the median *a*-edge; however, the added accuracy was not worth the computational cost of simulating each energy in the spectrum separately. This is why we elected to use the reduced equation (16) to model the Bragg scattering. (*c*) Dimensionless mosaic parameters *m_s_*. The initial value of 13.7 resulted from analyzing the mosaic domain size distribution deduced by *DIALS* during indexing. The median value of *m* after the stage 1 refinement (9.92) differs from the ground truth by 0.08. We hypothesize that this is because the synthetic data included a mosaic texture distribution that increased the size of the Bragg spots in reciprocal space. Smaller values of *m* correspond to larger Bragg spots, hence the model is likely trying to compensate for the absence of any mosaic texture in equation (16). (*d*) Dimensionless scale factors *G_s_*. To compare with the ground truth, all scale factors *G_s_* were divided by a factor *k* ^2^ where *k* = 3.68 is the scale factor which minimizes *R*_GT_ shown in equation (28). Initially we let all *G_s_* be 10^6^, which when divided by *k* ^2^ gives a value of 7.4 × 10^4^ as indicated by the red bar. (*e*) Misorientation of each crystal from its ground truth. During actual XFEL data collection one can never know this quantity, but synthetic data provides a unique opportunity to use this quantity as a proxy for model accuracy. We approach the optimal model when all 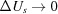. Note, the vertical axes in (*c*) and (*d*) and the horizontal axes in (*d*) and (*e*) are on logarithmic scales.

Initial estimates of the orientation matrices **U**_*s*_ and the crystal unit-cell matrices **B**_*s*_ were provided by the program *dials.stills_process* after successfully indexing each diffraction pattern (see Fig. 3 ▸) using the algorithm of Steller *et al.* (1997 ▸). The output from *dials.stills_process* also provided an estimate of the mosaic domain size for the measured crystals (Sauter *et al.*, 2014 ▸). We used these estimates to construct an initial guess of 13.7 for the mosaic parameters *m_s_*. The quantity 13.7 is the median mosaic domain size from *dials.stills_process* divided by the cubed root of the unit-cell volume, (*a* ^2^ *c*)^1/3^. The initial estimates for |*F*_**h**_| came from running the standard integration-based XFEL merging application in *CCTBX* (see Table 3 ▸), without post-refinement (see Table S1 of the supporting information for a comparison with post-refinement). The scale factors *G_s_* were each initially set to a very high number, in this case 10^6^. This number was chosen to ensure that the model initially predicted finite Bragg scattering in most of the shoeboxes. The background tilt-planes for all shoeboxes were initialized by solving equation (17) for each reflection shoebox.

### 3.2. Parameter estimation carried out in stages   

Once the parameters were initialized, maximum-likelihood parameter optimization was carried out in two main stages.

#### 3.2.1. Stage 1 refinement   

Here we refined shots one at a time in a series of two steps: first, for each shot, we refined the scale *G_s_* and the mosaic parameter *m_s_* while keeping all other parameters fixed. We did this using only the low-resolution shoeboxes (less than 5 Å) with signal-to-noise ratio greater than 3. Second, using the optimized values for *G_s_* and *m_s_*, the unit cell matrix **B**_*s*_ and the crystal rotation matrix **U**_*s*_ were refined while keeping all other parameters fixed. This was done for all spots with signal-to-noise ratio greater than 0.2 and resolution less than 2.1 Å. After this stage we identified images which refined poorly and removed them prior to the global stage 2 refinement. For the 2023 shot example, we removed 25 out of 2048 shots after stage 1 refinement by examining the resulting distributions for all *a_s_*, *c_s_, *m_s_** and *G_s_* (*a_s_* and *c_s_* are the unit-cell constants that fall out of the optimization of each **B**_*s*_). The results of stage 1 refinement are shown in Fig. 3 ▸ for 2023 exposures. For reparameterization in this stage of refinement we let σ_**U**_s__ = 0.001°, σ_**B**_s__ = 0.1 Å, σ_*G_s_*_ = 1 and σ_*m_s_*_ = 0.1 for all *s*, where σ_**U**_s__ corresponds to the expected variation in the three angles defining the crystal rotation matrices **U**_s_ and σ_**B_s_**_ corresponds to the expected variation in the unit-cell edge parameters. Timing statistics for this stage of refinement are shown in Table 4 ▸.

#### 3.2.2. Stage 2 refinement   

**Figure 4:**
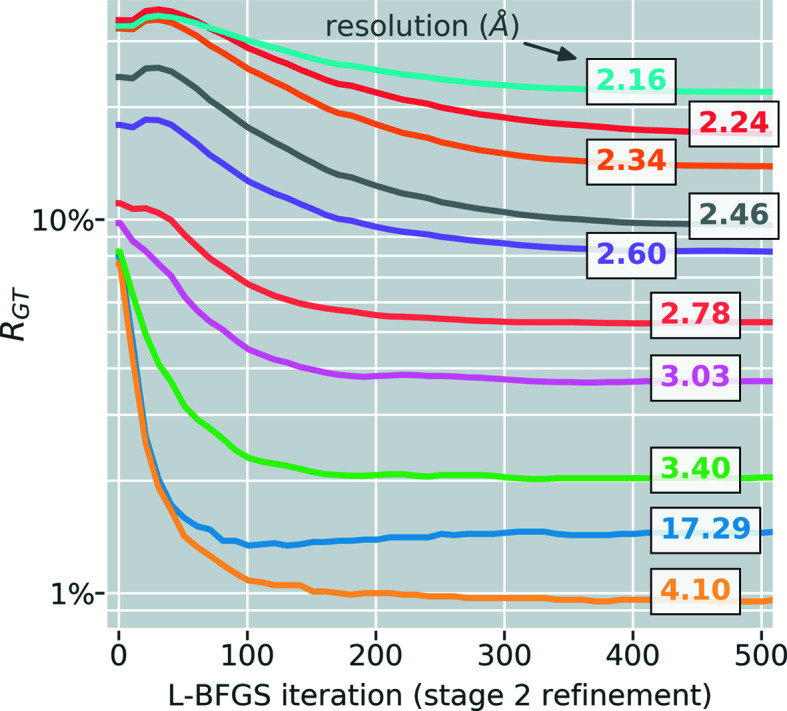
The *R* factor between refined structure factors and the ground truth (*R*_GT_) during stage 2 refinement.

Here we refined the structure factor amplitudes |*F*_**h**_| and the scale factors *G_s_* as part of a global refinement over all images. All other parameters were kept fixed. At the start of this refinement stage, we set all *m_s_* equal to the median of the values obtained during stage 1 [see Fig. 3 ▸(*c*)]. This was done because the per-shot scales *G_s_* and the per-shot mosaic domain parameters *m_s_* are correlated (both directly increase the number of expected photons in shot *s*). Also, at the start of this stage, all scale factors *G_s_* were set equal to the median of the results obtained for the scale factors during stage 1 refinement [see Fig. 3 ▸(*d*)], but then they optimized to different values for each shot. Fig. 4 ▸ illustrates stage 2 refinement results for the 2023 image set. This stage of refinement utilized all shoeboxes with a signal-to-noise ratio greater than 0.2 and resolution less than 2.1 Å. For reparameterization during this stage of refinement, we let σ_F_**h**__ = σ_G_s__ = 1 for all Miller indices **h** and shots *s.*

#### 3.2.3. Processing   

Standard message passing interface (MPI) was used to accelerate the analysis, parallelizing over images; however, beyond that no attempt was made to optimize the runtime. Timing tests for stage 1 and stage 2 refinement were conducted on a single compute node at NERSC comprising two 2.4 GHz Intel Xeon Gold processors for a total of 40 hardware threads. The complete set of data for all 2023 shots comprises 1.17 × 10^6^ Bragg spot shoeboxes for a total of 2.7 × 10^8^ pixels. For 2023 shots, stage 1 refinement (including input/output overhead) was completed in 12 min, utilizing all 40 hardware threads. Stage 2 refinement ran at a rate of 23.5 s per L-BFGS iteration using all 40 hardware threads (40 MPI ranks), though this number can be decreased by utilizing multiple compute nodes simultaneously. Future work will also utilize GPU-acceleration. The *nanoBragg* program used to compute the pixel values in equation (3) includes a GPU kernel which for our specific usen case currently offers a 528-fold speed-up over the CPU code, so we expect similar speed-ups for the minimization. Thus far, however, the methods used by *diffBragg* to compute the log-likelihood and its derivatives have only been written for CPUs.

### 3.3. Comparison of optimized parameters with ground truth values   

We judged the success of the *diffBragg* maximum-likelihood estimation by comparing refined parameters with the ground truth parameters used to synthesize the data. In this section we discuss three metrics important for judging the success of the refinement, namely the per-shot lattice misorientation with respect to the ground truth, the ground truth *R* factor and the anomalous difference correlation with the ground truth.

#### 3.3.1. Misorientation Δ*U_s_*   

A useful metric to observe during optimization is the misorientation Δ*U_s_* of the optimized crystal rotation matrices **U**_*s*_ from the known crystal rotation matrices used to synthesize each shot. Fig. 3 ▸(*e*) shows Δ*U_s_* before and after stage 1 refinement. For 2023 exposures, starting with a median Δ*U_s_* of 0.038° (as given by the *DIALS* indexing results), we were able to refine the crystal orientations such that the median Δ*U_s_* was 0.0028° [Fig. 3 ▸(*e*)].

#### 3.3.2. *R* factor with ground truth   

During optimization we monitored the *R* factor between the refined structure factor amplitudes and the ground truth (GT) structure factor amplitudes:
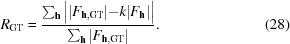
Here, *k* is a scale factor chosen to minimize *R*_GT_. Fig. 4 ▸ shows the evolution of *R*_GT_ throughout stage 2 refinement for different resolution bins. At each point in refinement we computed a new *k* using the Adaptive Nelder–Mead Simplex method (Gao & Han, 2012 ▸) as implemented in the *SciPy* software for Python.

#### 3.3.3. Anomalous difference correlation with ground truth   

**Figure 5:**
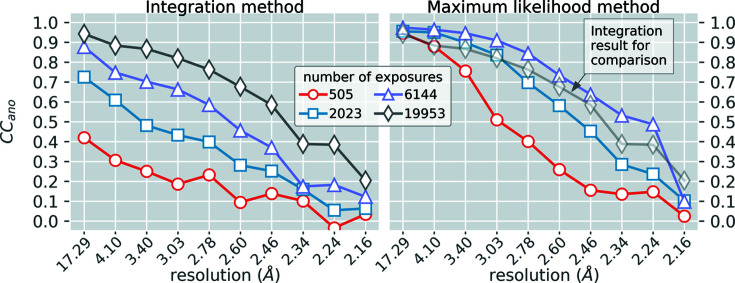
Comparison of 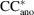 obtained during integration (left) with that obtained using the maximum-likelihood approach (right). 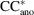 is directly proportional to the average peak height in an anomalous difference map at the positions of the heavy atoms which underwent significant X-ray absorption (Terwilliger *et al.*, 2016 ▸). It is simply the Pearson correlation coefficient between the observed difference structure factor amplitudes 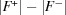 and the ground truth 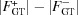, with the special requirement that *F*_GT_ only includes anomalous contributions from the absorptive heavy atoms (all other sources of anomalous scattering are therefore considered noise). In the right panel we show the integration result from 19 953 shots (faded gray diamonds) as a comparison with the maximum-likelihood results.

Because we targeted Yb^3+^ bound to lysozyme, we expected a strong anomalous component to be present. To this end we observed the correlation of anomalous difference amplitudes 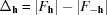 with those from the ground truth 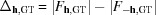. Specifically, we computed the Pearson correlation coefficient 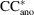 between 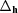 and 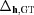. The correlation 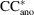 is discussed in great detail in the work by Terwilliger *et al.* (2016 ▸) where it was shown to be directly proportional to the peak height at sites of the absorptive heavy atoms in an anomalous difference density map, making it a good indicator of one’s ability to solve a SAD dataset. Note, it is common practice to report 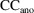 when discussing real data where one cannot know the ground truth model. This is accomplished using an empirical relationship outlined in the work by Terwilliger *et al.* (2016 ▸). Here, however, we are explicitly computing 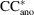 according to a ground truth model, hence the use of an asterisk in the defining symbol. Fig. 5 ▸ shows 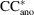 versus resolution for both the integration method and the maximum-likelihood method, where it is obvious that 2023 shots using maximum likelihood is comparable to 19 953 shots using integration. Overall values of *R*_GT_ and 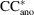 for various trials are shown in Table 5 ▸, for both the integration method and the maximum-likelihood method. With only 2023 exposures we achieved overall values of *R*_GT_ and 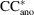 equal to 4.9% and 79%, respectively. Using the integration approach on the same images we got values of 11.0% and 48.4%, respectively. It is noteworthy that additional cycles of stage 1 and stage 2 provided little improvement beyond the initial cycle (Fig. S3).

## 4. Discussion   

The ability to accurately determine protein structure factor amplitudes |*F*_**h**_| in the presence of large experimental uncertainties largely governs the success of an SFX experiment. The maximum-likelihood program presented here, *diffBragg*, provides a direct protocol for decoupling the various contributions to the scattering which would otherwise obscure the structure factor amplitudes. These noisy contributions include variable per-shot incident photon spectra, crystal morphology and Ewald offset (partiality), all of which can be detrimental in situations involving weak signals, such as anomalous difference amplitudes used for the spatial resolution of heavy atoms (Sauter *et al.*, 2020 ▸), or for experimental phasing (Schlichting, 2017 ▸). With 2023 shots we achieved similar quality anomalous differences (revealed by 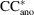) to those obtained by the conventional processing of 19 953 shots. Our approach eliminates the two-step process of measuring Bragg spot intensities on individual images, followed by merging. Rather, we refine the structure factors themselves against the raw data, in a restrained manner along with all other model parameters, to arrive at a stable solution. Also, the pixel-based approach provides more terms (one for each pixel) with which to restrain the optimization of the structure factor amplitudes, with the maximum-likelihood based optimization implicitly incorporating an error-based weighting scheme derived from the physical interpretation of signal measurement.

On a single compute node, refinement of 2023 shots took approximately 208 min, including 12 min for per-shot (stage 1) refinement and 196 min for 500 iterations of global (stage 2) refinement running at 23.5 s per iteration (Fig. 4 ▸ shows how structure factor quality improves with each iteration). On the same compute node, it took 157 s to index and integrate 2023 images with conventional methods, which were used to initialize the maximum-likelihood optimization. Therefore, the strategy going forward is to reduce this 75× wall-clock disparity by applying GPU acceleration to the maximum-likelihood step. We note that several beamline facilities, including LCLS, offer GPU-accelerated servers, with which it may be possible to run conventional integration and *diffBragg* in parallel with data collection, to better gauge experimental progress. We also plan to incorporate *diffBragg* as part of a broader effort centered on enabling leadership-class computing for rapid analysis of XFEL data. New compute facilities such as the pre-exascale system at NERSC (Perlmutter) and exascale systems at Argonne National Laboratory and Oak Ridge National Laboratory (Aurora and Frontier, respectively) will provide data processing at speeds to match the increased data collection rates expected for next-generation XFEL facilities (LCLS-II). The need for this level of computing (which includes GPU acceleration) becomes apparent by recalling equation (16), where we replaced the polychromatic spectrum of each shot with a simplified version. By instead including, for example, 100 energy channels for polychromatic model refinement, the 2023 shot refinement would take approximately 100 times longer, or 2350 s per iteration on a single 40-core CPU compute node.

We clarify that both stage 1 and stage 2 modeling are necessary to maximize the information extracted from the data. To illustrate this, we used the results from stage 1 refinement to compute model-derived partiality corrections for each integrated spot. By correcting each integrated spot intensity using these new terms, we were able to increase 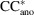 beyond that obtained with uncorrected integrated intensities; however, stage 2 refinement consistently yielded the best results (see Section S2 and Table S2). For example, using 2023 shots we were able to boost 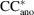 to 0.57: an improvement over uncorrected integration, yet still worse than the value of 0.79 obtained with *diffBragg*.

It is common to model the aggregate effect of energy-bandwidth and mosaicity in a single Gaussian equation (Parkhurst, 2020 ▸, Chapter 5), yet *diffBragg* is a general framework. Notably, the work shown is directly applicable to two-color serial diffraction (Hara *et al.*, 2013 ▸; Lu *et al.*, 2018 ▸; Lutman *et al.*, 2014 ▸) and pink-beam serial diffraction (Dejoie *et al.*, 2013 ▸; Milne *et al.*, 2017 ▸; Martin-Garcia *et al.*, 2019 ▸) where indexing, refinement and data reduction protocols are in early development (Gevorkov *et al.*, 2020 ▸). The work presented here assumed a perfect detector geometry; however, we demonstrated the robustness of *diffBragg* refinement to typical levels of detector panel displacement (see Section S3, Figs. S4 and S5, and Table S3). We also assumed a detector with minimal pixel crosstalk and a well calibrated linear response, attributes that are realized in current-generation detectors like the ePix (Sikorski *et al.*, 2016 ▸), JUNGFRAU (Leonarski *et al.*, 2018 ▸) and AGIPD (Allahgholi *et al.*, 2015 ▸). On the other hand, significant point-spread occurs in detectors such as the widely used Rayonix (Holton *et al.*, 2012 ▸; Ke *et al.*, 2018 ▸). Using results derived in the work by Holton *et al.* (2012 ▸), we applied a point-spread function to the synthesized data and observed that structure factor optimization could still proceed, provided the point-spread kernel was also applied to the model *n*_*i*,*s*_(Θ) (see Section S4, and Figs. S6 and S7). Sources of error we have not described include measurement parallax, per-image Debye–Waller factors, intra-crystal unit-cell variation and multiple lattice scattering, all of which contribute to error in the determined structure factors. Thus far we have neglected any mention of structure factor errors, but we know proper error estimation can aid in solving real systems (Brewster, Bhowmick *et al.*, 2019 ▸). In order to obtain error estimates for the structure factor amplitudes in the current context, one can consider the second derivative of the log-likelihood expression evaluated at the maximum-likelihood estimate:
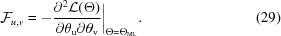
Here, 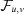 is called the Fisher information matrix, 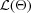 is the log-likelihood defined in equation (22), and *u* and *v* are row and column indices. The variances of the parameters Θ_ML_, including the structure factors {|*F*_**h**_|}, are given by the diagonal elements of 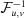 (Pawitan, 2001 ▸). The matrix 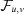 is large, and inversion should be performed using sparse matrix algebra. Our future efforts will involve implementing this computation.

In summary, we used the program *nanoBragg* to generate realistic XFEL diffraction images, which we analyzed using both the standard integration protocol and the new program, *diffBragg*. With *diffBragg*, we utilized all measured pixels simultaneously to estimate high-accuracy structure factors while requiring an order of magnitude fewer diffraction events compared with the conventional method. Reducing the number of required shots for a dataset can greatly benefit the general SFX experiment, with scarce beam time routinely plagued by unforeseen interruptions, limiting the amount of data one can realistically collect. Future work will aim to apply this method to real measurements and develop a user-friendly application for the general SFX researcher.

## 6. Software availability   

The tools used for *diffBragg* refinement are included in *CCTBX*. Scripts for computing and processing the synthetic data are available at https://github.com/dermen/cxid9114.

## Supplementary Material

Supporting figures and tables detailing the effects of mosaic spread and geometry errors on the refinement. DOI: 10.1107/S2052252520013007/zf5012sup1.pdf

## Figures and Tables

**Table 1:** Parameters describing the expected number of photons scattered into each pixel

κ_*i*_	Kahn polarization factor
*r*_e_	Classical electron radius
*J_s_*(λ)	Fluence (photons/area) at photon wavelength λ during shot *s*
Δ**h**_*i*,*j*,*s*_	Ewald offset of pixel *i* for scattering from mosaic domain *j* during shot *s*, *i.e.* the residual between the fractional Miller index and the integer Miller index **h** = *h*, *k*, *l*
*I*_0_(Δ**h**_*i*,*j*,*s*_, *m*)	The interference function arising from the diffraction of the crystal lattice
ΔΩ_*i*_	The solid angle subtended by the pixel
*Z_s_*	Dimensionless per-shot scale factor accounting for variations in exposed crystal volume for each shot. This represents the total number of mosaic domains in the crystal
*m*	Dimensionless quantity such that *m* ^3^ specifies the number of unit cells within each mosaic domain. For the synthetic data, we let *m* = 10, hence we used mosaic blocks consisting of 1000 unit cells
|*F*_**h**_|	Structure factor amplitude of the protein at Miller index **h** = *h*, *k*, *l*
λ	Photon wavelength
**U**_*j*,*s*_	Misseting matrix of mosaic domain *j* for the crystal from shot *s*
**B**	Direct space unit cell matrix for each crystal (same unit cell for each crystal)
*N_j_*	Number of mosaic domains modeling texture. For the synthetic data, we let *N_j_* = 100
**q**_*i*_(λ)	Momentum transfer vector at pixel *i* for photons of wavelength λ

**Table 2:** Summary of all model parameters that are refined in order to maximize the likelihood of the data

*G_s_*	Scale factor for the Bragg scattering observed during shot *s*
**U**_*s*_	Missetting matrix of the crystal measured during shot *s*
*m_s_*	Cube root of the number of unit cells in each mosaic domain
**B**_*s*_	Crystal unit-cell matrix for shot *s*
{|*F*_**h**_|}	Full set of protein structure factor amplitudes

**Table 3:** The integration-based merging statistics from *CCTBX* from 2023 synthetic shots

Resolution bin range (Å)	Completeness (%)	Multiplicity	Measurements	〈*I*/σ_*I*_〉	CC_1/2_ (%)	*R*-split (%)
30.00–4.573	100	104.12	137235	16.65	99.6	6.0
4.573–3.632	100	64.06	83988	13.01	97.7	8.7
3.632–3.174	100	47.26	61766	10.40	97.5	9.9
3.174–2.884	100	44.69	58404	9.29	96.7	11.4
2.884–2.677	100	40.34	53131	8.00	95.5	12.8
2.677–2.519	100	36.01	47250	7.04	93.8	14.8
2.519–2.393	100	37.40	49036	6.87	90.7	15.6
2.393–2.289	100	35.77	47147	5.97	88.6	18.6
2.289–2.200	100	27.90	36324	4.50	79.7	25.2
2.200–2.125	100	23.17	30307	3.23	71.3	34.4
Overall	100	46.11	604588	8.50	99.6	9.1

**Table 4:** Stage 1 refinement CPU usage at NERSC

		Average processing time per image (core-seconds)	
Refinement level	Average iterations per image	2.4 GHz Intel Xeon Gold 6148	2.3 GHz Intel Xeon E5-2698 v3
Stage 1, part 1: *G_s_* and *m_s_*	17.02	2.12	2.53
Stage 1, part 2: **U**_*s*_ and **B**_*s*_	6.54	7.04	8.42

**Table 5:** Overall structure factor quality for various trials (completeness for each merge is 100% out to a maximum resolution of 2.125 Å)

	Integration		Maximum likelihood		
No. of exposures	*R*_GT_	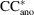	*R*_GT_	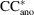	No. of L-BFGS iterations (stage 2)
505	0.140	0.230	0.059	0.479	576
2023	0.110	0.484	0.049	0.790	528
6144	0.105	0.696	0.049	0.904	359
19953	0.104	0.856	N/A	N/A	N/A

## Footnotes

1 Recently, ‘whole pattern’ methods have demonstrated the fitting of entire serial diffraction datasets to a global three-dimensional intensity model (Dilanian *et al.*, 2016 ▸; Lan *et al.*, 2018 ▸), avoiding the integration step completely. Lan *et al.* (2018 ▸) employed the expand–maximize–compress (EMC) algorithm (Loh & Elser, 2009 ▸), derived for single-particle imaging, to process serial millisecond crystallography data. By employing the maximum-likelihood approach, they extracted structure factor intensities from weak signals in the context of a stable synchrotron source.

## Appendix A

The interference term *I*_0_ for a parallelepiped mosaic block consisting of *N_a_* × *N_b_* × *N_c_* unit cells stacked along the crystal axes *a*, *b*, *c* is given in the work by James (1962 ▸),

where the subscript *f* is used to indicate a fractional Miller index evaluated at a pixel. *I*_0_ exhibits principal maxima at integers *h*, *k*, *l* and subsidiary maxima along lines between *h*, *k*, *l* grid points as observed in the work by Chapman *et al.* (2011 ▸). As *I*_0_ is the modulus squared of the Fourier transform of all unit-cell origins in the mosaic block, each principal maximum is equal to the squared number of unit cells (*N_a_* *N_b_* *N_c_*)^2^. One can verify this claim by applying L’Hôpital’s rule to find the limit of equation (30) as (*h_f_*, *k_f_*, *l_f_*) tends to (0, 0, 0). We wish to model the principal peaks in *I*_0_ using Gaussians, but for that we also have to analyze the full width at half-maximum, *W*, of the principal peaks. *W* can be found numerically by solving the transcendental equation

for *x* = *x*_fwhm_ along the interval −0.5 < *x* < 0.5, such that *W* = 2|*x*_fwhm_|. Numerically solving equation (31) for a range of *N* (5 ≤ *N* ≤ 100) reveals an inverse *N* dependence on *W*, as expected given the physical interpretation of *I*_0_ (larger crystals produce smaller reciprocal space peaks):

With this we can write the standard deviation of an appropriate Gaussian form as

hence, we can approximate

where Δ*h*, Δ*k*, Δ*l* represent the distance from each value *h_f_*, *k_f_*, *l_f_* to its nearest integer value *h*, *k*, *l* (*e.g.* Δ*h* = *h_f_* − *h*). Substituting equation (33) into (34) we arrive at

Letting *N_a_* = *N_b_* = *N_c_* = *m* brings us to the form shown in equation (4), with *C* = 1/0.286 (though we actually used *C* = 2/0.63 during all of this work as that was the default parameter in *nanoBragg*). The precise value of the constant in equation (35) can change for different lattices, and one can potentially refine it along with the mosaic parameter *m* for each lattice in order to obtain a better fit to the data. The Gaussian approximation is useful computationally as it lends itself to simpler derivatives, while also retaining the main properties of the analytical expression in equation (30).

## Appendix B

*Scale factor derivative.* We define its derivative as

The scale factor *G_s_* should always be a positive quantity, therefore to each *G_s_* we apply a reparameterization of the form , such that  (after applying the chain rule). This ensures the scale never becomes negative during refinement.

**U** *_s_* *-matrix derivative.* In practice, rather than minimizing the absolute misorientation angles in the matrix **U**_*s*_(ϕ_*x*_, ϕ_*y*_, ϕ_*z*_), we instead define three refinement parameters representing angles about the standard laboratory-frame basis vectors, *e.g.* for the laboratory (lab) *x* axis we define the rotation operator

We define similar matrices for the laboratory *y* and *z* axes. The matrix **U** *_s_* is then redefined as **U**_*s*_ = **R**_*x*_ **R**_*y*_ **R**_*z*_ **U**_*s*,o_ where **U**_*s*,o_ is the initial unitary matrix determined by the indexing protocol and then held fixed during refinement. We refine the parameters , after initializing them all to 0. The Ewald offset  is now redefined as , where **h** = (*h*, *k*, *l*) is the integer Miller index, and the derivative of the expected scattered photons with respect to the angular offset parameter is given by

where

and *C* is the parameter describing the lattice transform (see Appendix *A* [App appa]). Similar expressions follow for  and . No restraints were applied to the rotation angles during refinement, hence the reparameterizations used were of the form (1/σ_Δϕ_)Δϕ + 1.

**B** *-matrix derivative.* The derivative of the mean scattered photons with respect to the unit-cell matrix is similar to that derived for **U**_*s*_. We show here the case for the unit cell *a* edge:

where

A similar result follows for the *c* edge. Reparameterization could be applied in order to keep *a* and *c* within a valid range; however, we never encountered a situation where *a* or *c* diverged. We therefore applied reparameterizations of the form *x*_*a*_ = (1/σ_*a*_)(*a* − *a*_o_) + 1.

*Derivative of mosaic parameter m.* The derivative of the mean scattered photons with respect to *m* is given by

For this parameter we performed a reparameterization to ensure that *m* was always greater than 3, hence we use the reparameterization *x*_*m*_ = (1/σ_*m*_)[ln(*m* − 3) − ln(*m*_o_ − 3)] + 1.

## References

[bb1] Allahgholi, A., Becker, J., Bianco, L., Delfs, A., Dinapoli, R., Goettlicher, P., Graafsma, H., Greiffenberg, D., Hirsemann, H., Jack, S., Klanner, R., Klyuev, A., Krueger, H., Lange, S., Marras, A., Mezza, D., Mozzanica, A., Rah, S., Xia, Q., Schmitt, B., Schwandt, J., Sheviakov, I., Shi, X., Smoljanin, S., Trunk, U., Zhang, J. & Zimmer, M. (2015). *J. Instrum.* **10**, C01023.

[bb3] Alonso-Mori, R., Asa, K., Bergmann, U., Brewster, A. S., Chatterjee, R., Cooper, J. K., Frei, H. M., Fuller, F. D., Goggins, E., Gul, S., Fukuzawa, H., Iablonskyi, D., Ibrahim, M., Katayama, T., Kroll, T., Kumagai, Y., McClure, B. A., Messinger, J., Motomura, K., Nagaya, K., Nishiyama, T., Saracini, C., Sato, Y., Sauter, N. K., Sokaras, D., Takanashi, T., Togashi, T., Ueda, K., Weare, W. W., Weng, T. C., Yabashi, M., Yachandra, V. K., Young, I. D., Zouni, A., Kern, J. F. & Yano, J. (2016). *Faraday Discuss.* **194**, 621–638. 10.1039/c6fd00084c PMC5177497 27711803

[bb5] Arndt, U. W. & Wonacott, A. J. (1977). *The Rotation Method in Crystallography.* Amsterdam: North-Holland.

[bb7] Azároff, L. V. (1955). *Acta Cryst.* **8**, 701–704.

[bb9] Bellamy, H. D., Snell, E. H., Lovelace, J., Pokross, M. & Borgstahl, G. E. O. (2000). *Acta Cryst.* D**56**, 986–995. 10.1107/s0907444900007356 10944335

[bb11] Brewster, A. S., Bhowmick, A., Bolotovsky, R., Mendez, D., Zwart, P. H. & Sauter, N. K. (2019). *Acta Cryst.* D**75**, 959–968. 10.1107/S2059798319012877 PMC6834081 31692470

[bb13] Brewster, A. S., Waterman, D. G., Parkhurst, J. M., Gildea, R. J., Michels-Clark, T. M., Young, I. D., Bernstein, H. J., Winter, G., Evans, G. & Sauter, N. K. (2016). *Comput. Crystallogr. Newslett.* **7**, 32–53.

[bb15] Brewster, A. S., Waterman, D. G., Parkhurst, J. M., Gildea, R. J., Young, I. D., O’Riordan, L. J., Yano, J., Winter, G., Evans, G. & Sauter, N. K. (2018). *Acta Cryst.* D**74**, 877–894. 10.1107/S2059798318009191 PMC6130462 30198898

[bb17] Brewster, A. S., Young, I. D., Lyubimov, A., Bhowmick, A. & Sauter, N. K. (2019). *Comput. Crystallogr. Newslett.* **10**, 22–39.

[bb19] Brown, P. J., Fox, A. G., Maslen, E. N., O’Keefe, M. A. & Willis, B. T. M. (2006). *International Tables for Crystallography*, Vol. C, 1st online ed., Section 6.1.1, pp. 554–590. Chester: International Union of Crystallography.

[bb21] Busing, W. R. & Levy, H. A. (1967). *Acta Cryst.* **22**, 457–464.

[bb23] Chapman, H. N., Caleman, C. & Timneanu, N. (2014). *Philos. Trans. R. Soc. B*, **369**, 20130313. 10.1098/rstb.2013.0313 PMC4052855 24914146

[bb26] Chapman, H. N., Fromme, P., Barty, A., White, T. A., Kirian, R. A., Aquila, A., Hunter, M. S., Schulz, J., DePonte, D. P., Weierstall, U., Doak, R. B., Maia, F. R. N. C., Martin, A. V., Schlichting, I., Lomb, L., Coppola, N., Shoeman, R. L., Epp, S. W., Hartmann, R., Rolles, D., Rudenko, A., Foucar, L., Kimmel, N., Weidenspointner, G., Holl, P., Liang, M., Barthelmess, M., Caleman, C., Boutet, S., Bogan, M. J., Krzywinski, J., Bostedt, C., Bajt, S., Gumprecht, L., Rudek, B., Erk, B., Schmidt, C., Hömke, A., Reich, C., Pietschner, D., Strüder, L., Hauser, G., Gorke, H., Ullrich, J., Herrmann, S., Schaller, G., Schopper, F., Soltau, H., Kühnel, K., Messerschmidt, M., Bozek, J. D., Hau-Riege, S. P., Frank, M., Hampton, C. Y., Sierra, R. G., Starodub, D., Williams, G. J., Hajdu, J., Timneanu, N., Seibert, M. M., Andreasson, J., Rocker, A., Jönsson, O., Svenda, M., Stern, S., Nass, K., Andritschke, R., Schröter, C., Krasniqi, F., Bott, M., Schmidt, K. E., Wang, X., Grotjohann, I., Holton, J. M., Barends, T. R. M., Neutze, R., Marchesini, S., Fromme, R., Schorb, S., Rupp, D., Adolph, M., Gorkhover, T., Andersson, I., Hirsemann, H., Potdevin, G., Graafsma, H., Nilsson, B. & Spence, J. C. H. (2011). *Nature*, **470**, 73–77.

[bb27] Clark, G. N. I., Hura, G. L., Teixeira, J., Soper, A. K. & Head-Gordon, T. (2010). *Proc. Natl Acad. Sci. USA*, **107**, 14003–14007. 10.1073/pnas.1006599107 PMC2922569 20647388

[bb29] Darwin, C. G. (1922). *London Edinb. Dubl. Philos. Mag. J. Sci.* **43**, 800–829.

[bb31] Dasgupta, M., Budday, D., de Oliveira, S. H. P., Madzelan, P., Marchany-Rivera, D., Seravalli, J., Hayes, B., Sierra, R. G., Boutet, S., Hunter, M. S., Alonso-Mori, R., Batyuk, A., Wierman, J., Lyubimov, A., Brewster, A. S., Sauter, N. K., Applegate, G. A., Tiwari, V. K., Berkowitz, D. B., Thompson, M. C., Cohen, A. E., Fraser, J. S., Wall, M. E., van den Bedem, H. & Wilson, M. A. (2019). *Proc. Natl Acad. Sci. USA*, **116**, 25634–25640. 10.1073/pnas.1901864116 PMC6926069 31801874

[bb33] Dejoie, C., McCusker, L. B., Baerlocher, C., Abela, R., Patterson, B. D., Kunz, M. & Tamura, N. (2013). *J. Appl. Cryst.* **46**, 791–794.

[bb35] DePonte, D. P., Weierstall, U., Schmidt, K., Warner, J., Starodub, D., Spence, J. C. H. & Doak, R. B. (2008). *J. Phys. D Appl. Phys.* **41**, 195505.

[bb37] Diederichs, K. (2009). *Acta Cryst.* D**65**, 535–542. 10.1107/S0907444909010282 19465767

[bb39] Dilanian, R. A., Williams, S. R., Martin, A. V., Stretsov, V. A. & Quiney, H. M. (2016). *IUCrJ*, **3**, 127–138. 10.1107/S2052252516001238 PMC4775161 27006776

[bb41] Duisenberg, A. J. M., Kroon-Batenburg, L. M. J. & Schreurs, A. M. M. (2003). *J. Appl. Cryst.* **36**, 220–229.

[bb43] Emma, P., Bane, K., Cornacchia, M., Huang, Z., Schlarb, H., Stupakov, G. & Walz, D. (2004). *Phys. Rev. Lett.* **92**, 074801. 10.1103/PhysRevLett.92.074801 14995861

[bb45] Evans, P. R. & Murshudov, G. N. (2013). *Acta Cryst.* D**69**, 1204–1214. 10.1107/S0907444913000061 PMC3689523 23793146

[bb47] Fransson, T., Chatterjee, R., Fuller, F. D., Gul, S., Weninger, C., Sokaras, D., Kroll, T., Alonso-Mori, R., Bergmann, U., Kern, J., Yachandra, V. K. & Yano, J. (2018). *Biochemistry*, **57**, 4629–4637. 10.1021/acs.biochem.8b00325 PMC6081253 29906115

[bb49] Gao, F. & Han, L. (2012). *Comput. Optim. Appl.* **51**, 259–277.

[bb51] Gevorkov, Y., Barty, A., Brehm, W., White, T. A., Tolstikova, A., Wiedorn, M. O., Meents, A., Grigat, R.-R., Chapman, H. N. & Yefanov, O. (2020). *Acta Cryst.* A**76**, 121–131. 10.1107/S2053273319015559 PMC7053222 32124850

[bb53] Ginn, H. M., Evans, G., Sauter, N. K. & Stuart, D. I. (2016). *J. Appl. Cryst.* **49**, 1065–1072. 10.1107/S1600576716006981 PMC4886992 27275149

[bb55] Grosse-Kunstleve, R. W., Sauter, N. K., Moriarty, N. W. & Adams, P. D. (2002). *J. Appl. Cryst.* **35**, 126–136.

[bb57] Hammersley, A. (2009). *FIT2D*. ESRF, Grenoble, France.

[bb59] Hara, T., Inubushi, Y., Katayama, T., Sato, T., Tanaka, H., Tanaka, T., Togashi, T., Togawa, K., Tono, K., Yabashi, M. & Ishikawa, T. (2013). *Nat. Commun.* **4**, 1–5. 10.1038/ncomms3919 24301682

[bb61] Hart, P., Boutet, S., Carini, G., Dubrovin, M., Duda, B., Fritz, D., Haller, G., Herbst, R., Herrmann, S., Kenney, C., Kurita, N., Lemke, H., Messerschmidt, M., Nordby, M., Pines, J., Schafer, D., Swift, M., Weaver, M., Williams, G., Zhu, D., Van Bakel, N. & Morse, J. (2012). *Proc. SPIE*, **8504**, 85040C.

[bb900] Hendrickson, W. A. & Ogata, C. M. (1997). *Methods Enzymol.* **276**, 494–523. 10.1016/S0076-6879(97)76074-9 27799111

[bb63] Henke, B. L., Gullikson, E. M. & Davis, J. C. (1993). *At. Data Nucl. Data Tables*, **54**, 181–342.

[bb65] Holton, J. M., Classen, S., Frankel, K. A. & Tainer, J. A. (2014). *FEBS J.* **281**, 4046–4060. 10.1111/febs.12922 PMC4282448 25040949

[bb67] Holton, J. M. & Frankel, K. A. (2010). *Acta Cryst.* D**66**, 393–408. 10.1107/S0907444910007262 PMC2852304 20382993

[bb69] Holton, J. M., Nielsen, C. & Frankel, K. A. (2012). *J. Synchrotron Rad.* **19**, 1006–1011. 10.1107/S0909049512035571 PMC3480276 23093762

[bb72] Ibrahim, M., Fransson, T., Chatterjee, R., Cheah, M. H., Hussein, R., Lassalle, L., Sutherlin, K. D., Young, I. D., Fuller, F. D., Gul, S., Kim, I. S., Simon, P. S., de Lichtenberg, C., Chernev, P., Bogacz, I., Pham, C. C., Orville, A. M., Saichek, N., Northen, T., Batyuk, A., Carbajo, S., Alonso-Mori, R., Tono, K., Owada, S., Bhowmick, A., Bolotovsky, R., Mendez, D., Moriarty, N. W., Holton, J. M., Dobbek, H., Brewster, A. S., Adams, P. D., Sauter, N. K., Bergmann, U., Zouni, A., Messinger, J., Kern, J., Yachandra, V. K. & Yano, J. (2020). *Proc. Natl Acad. Sci. USA*, **117**, 12624–12635.

[bb73] James, R. W. (1962). *The Optical Principles of the Diffraction of X-rays*. London: Bell.

[bb75] Kabsch, W. (2014). *Acta Cryst.* D**70**, 2204–2216. 10.1107/S1399004714013534 PMC4118830 25084339

[bb77] Kahn, R., Fourme, R., Gadet, A., Janin, J., Dumas, C. & André, D. (1982). *J. Appl. Cryst.* **15**, 330–337.

[bb79] Ke, T.-W., Brewster, A. S., Yu, S. X., Ushizima, D., Yang, C. & Sauter, N. K. (2018). *J. Synchrotron Rad.* **25**, 655–670. 10.1107/S1600577518004873 PMC5929353 29714177

[bb81] Kern, J., Chatterjee, R., Young, I. D., Fuller, F. D., Lassalle, L., Ibrahim, M., Gul, S., Fransson, T., Brewster, A. S., Alonso-Mori, R., Hussein, R., Zhang, M., Douthit, L., de Lichtenberg, C., Cheah, M. H., Shevela, D., Wersig, J., Seuffert, I., Sokaras, D., Pastor, E., Weninger, C., Kroll, T., Sierra, R. G., Aller, P., Butryn, A., Orville, A. M., Liang, M., Batyuk, A., Koglin, J. E., Carbajo, S., Boutet, S., Moriarty, N. W., Holton, J. M., Dobbek, H., Adams, P. D., Bergmann, U., Sauter, N. K., Zouni, A., Messinger, J., Yano, J. & Yachandra, V. K. (2018). *Nature*, **563**, 421–425.

[bb83] Kirian, R. A., Wang, X., Weierstall, U., Schmidt, K. E., Spence, J. C. H., Hunter, M., Fromme, P., White, T., Chapman, H. N. & Holton, J. (2010). *Opt. Express*, **18**, 5713–5723. 10.1364/OE.18.005713 PMC4038330 20389587

[bb85] Kroon-Batenburg, L. M. J., Schreurs, A. M. M., Ravelli, R. B. G. & Gros, P. (2015). *Acta Cryst.* D**71**, 1799–1811. 10.1107/S1399004715011803 PMC4556312 26327370

[bb87] Lan, T.-Y., Wierman, J. L., Tate, M. W., Philipp, H. T., Martin-Garcia, J. M., Zhu, L., Kissick, D., Fromme, P., Fischetti, R. F., Liu, W., Elser, V. & Gruner, S. M. (2018). *IUCrJ*, **5**, 548–558. 10.1107/S205225251800903X PMC6126656 30224958

[bb90] Leonarski, F., Redford, S., Mozzanica, A., Lopez-Cuenca, C., Panepucci, E., Nass, K., Ozerov, D., Vera, L., Olieric, V., Buntschu, D., Schneider, R., Tinti, G., Froejdh, E., Diederichs, K., Bunk, O., Schmitt, B. & Wang, M. (2018). *Nat. Methods*, **15**, 799–804. 10.1038/s41592-018-0143-7 30275593

[bb92] Leslie, A. G. W. (1999). *Acta Cryst.* D**55**, 1696–1702. 10.1107/s090744499900846x 10531519

[bb94] Liang, M., Williams, G. J., Messerschmidt, M., Seibert, M. M., Montanez, P. A., Hayes, M., Milathianaki, D., Aquila, A., Hunter, M. S., Koglin, J. E., Schafer, D. W., Guillet, S., Busse, A., Bergan, R., Olson, W., Fox, K., Stewart, N., Curtis, R., Miahnahri, A. A. & Boutet, S. (2015). *J. Synchrotron Rad.* **22**, 514–519. 10.1107/S160057751500449X PMC4416669 25931062

[bb96] Liu, D. C. & Nocedal, J. (1989). *Math. Program.* 45, 503–528.

[bb98] Loh, N. D. & Elser, V. (2009). *Phys. Rev. E*, **80**, 026705. 10.1103/PhysRevE.80.026705 19792279

[bb100] Lu, W., Friedrich, B., Noll, T., Zhou, K., Hallmann, J., Ansaldi, G., Roth, T., Serkez, S., Geloni, G., Madsen, A. & Eisebitt, S. (2018). *Rev. Sci. Instrum.* **89**, 063121. 10.1063/1.5027071 29960553

[bb102] Lutman, A. A., Decker, F.-J., Arthur, J., Chollet, M., Feng, Y., Hastings, J., Huang, Z., Lemke, H., Nuhn, H.-D., Marinelli, A., Turner, J. L., Wakatsuki, S., Welch, J. & Zhu, D. (2014). *Phys. Rev. Lett.* **113**, 254801. 10.1103/PhysRevLett.113.254801 25554887

[bb104] Lyubimov, A. Y., Uervirojnangkoorn, M., Zeldin, O. B., Zhou, Q., Zhao, M., Brewster, A. S., Michels-Clark, T., Holton, J. M., Sauter, N. K., Weis, W. I. & Brunger, A T. (2016). *eLife*, **5**, e18740. 10.7554/eLife.18740 PMC5094853 27731796

[bb106] MacDowell, A. A., Celestre, R. S., Howells, M., McKinney, W., Krupnick, J., Cambie, D., Domning, E. E., Duarte, R. M., Kelez, N., Plate, D. W., Cork, C. W., Earnest, T. N., Dickert, J., Meigs, G., Ralston, C., Holton, J. M., Alber, T., Berger, J. M., Agard, D. A. & Padmore, H. A. (2004). *J. Synchrotron Rad.* **11**, 447–455. 10.1107/S0909049504024835 15496731

[bb108] Margaritondo, G. & Rebernik Ribic, P. (2011). *J. Synchrotron Rad.* **18**, 101–108. 10.1107/S090904951004896X PMC3042323 21335894

[bb110] Martin-Garcia, J. M., Zhu, L., Mendez, D., Lee, M.-Y., Chun, E., Li, C., Hu, H., Subramanian, G., Kissick, D., Ogata, C., Henning, R., Ishchenko, A., Dobson, Z., Zhang, S., Weierstall, U., Spence, J. C. H., Fromme, P., Zatsepin, N. A., Fischetti, R. F., Cherezov, V. & Liu, W. (2019). *IUCrJ*, **6**, 412–425. 10.1107/S205225251900263X PMC6503920 31098022

[bb112] Milne, C. J., Schietinger, T., Aiba, M., Alarcon, A., Alex, J., Anghel, A., Arsov, V., Beard, C., Beaud, P., Bettoni, S., Bopp, M., Brands, H., Brönnimann, M., Brunnenkant, I., Calvi, M., Citterio, A., Craievich, P., Csatari Divall, M., Dällenbach, M., D’Amico, M., Dax, A., Deng, Y., Dietrich, A., Dinapoli, R., Divall, E., Dordevic, S., Ebner, S., Erny, C., Fitze, H., Flechsig, U., Follath, R., Frei, F., Gärtner, F., Ganter, R., Garvey, T., Geng, Z., Gorgisyan, I., Gough, C., Hauff, A., Hauri, C. P., Hiller, N., Humar, T., Hunziker, S., Ingold, G., Ischebeck, R., Janousch, M., Juranić, P., Jurcevic, M., Kaiser, M., Kalantari, B., Kalt, R., Keil, B., Kittel, C., Knopp, G., Koprek, W., Lemke, H. T., Lippuner, T., Llorente Sancho, D., Löhl, F., Lopez-Cuenca, C., Märki, F., Marcellini, F., Marinkovic, G., Martiel, I., Menzel, R., Mozzanica, A., Nass, K., Orlandi, G. L., Ozkan Loch, C., Panepucci, E., Paraliev, M., Patterson, B., Pedrini, B., Pedrozzi, M., Pollet, P., Pradervand, C., Prat, E., Radi, P., Raguin, J.-Y., Redford, S., Rehanek, J., Réhault, J., Reiche, S., Ringele, M., Rittmann, J., Rivkin, L., Romann, A., Ruat, M., Ruder, C., Sala, L., Schebacher, L., Schilcher, T., Schlott, V., Schmidt, T., Schmitt, B., Shi, X., Stadler, M., Stingelin, L., Sturzenegger, W., Szlachetko, J., Thattil, D., Treyer, D. M., Trisorio, A., Tron, W., Vetter, S., Vicario, C., Voulot, D., Wang, M., Zamofing, T., Zellweger, C. & Zennaro, R. (2017). *Appl. Sci.* **7**, 720.

[bb114] Nango, E., Kubo, M., Tono, K. & Iwata, S. (2019). *Appl. Sci.* **9**, 5505.

[bb116] Nave, C. (1998). *Acta Cryst.* D**54**, 848–853. 10.1107/s0907444998001875 9757100

[bb118] Nave, C. (2014). *J. Synchrotron Rad.* **21**, 537–546. 10.1107/S1600577514003609 PMC3998814 24763643

[bb120] Nogly, P., Weinert, T., James, D., Carbajo, S., Ozerov, D., Furrer, A., Gashi, D., Borin, V., Skopintsev, P., Jaeger, K., Nass, K., Bath, P., Bosman, R., Koglin, J., Seaberg, M., Lane, T., Kekilli, D., Brunle, S., Tanaka, T., Wu, W., Milne, C., White, T., Barty, A., Weierstall, U., Panneels, V., Nango, E., Iwata, S., Hunter, M., Schapiro, I., Schertler, G., Neutze, R. & Standfuss, J. (2018). *Science*, **361**, eaat0094. 10.1126/science.aat0094 29903883

[bb122] Pande, K., Hutchison, C. D. M., Groenhof, G., Aquila, A., Robinson, J. S., Tenboer, J., Basu, S., Boutet, S., DePonte, D. P., Liang, M., White, T. A., Zatsepin, N. A., Yefanov, O., Morozov, D., Oberthuer, D., Gati, C., Subramanian, G., James, D., Zhao, Y., Koralek, J., Brayshaw, J., Kupitz, C., Conrad, C., Roy-Chowdhury, S., Coe, J. D., Metz, M., Xavier, P. L., Grant, T. D., Koglin, J. E., Ketawala, G., Fromme, R., rajer, V., Henning, R., Spence, J. C. H., Ourmazd, A., Schwander, P., Weierstall, U., Frank, M., Fromme, P., Barty, A., Chapman, H. N., Moffat, K., van Thor, J. J. & Schmidt, M. (2016). *Science*, **352**, 725–729.

[bb124] Parkhurst, J. M. (2020). *Statistically Robust Methods for the Integration and Analysis of X-ray Diffraction Data from Pixel Array Detectors*. Doctoral thesis, University of Cambridge.

[bb126] Pawitan, Y. (2001). *In All Likelihood: Statistical Modelling and Inference using Likelihood.* Oxford University Press.

[bb128] Pinker, F., Brun, M., Morin, P., Deman, A.-L., Chateaux, J.-F., Oliéric, V., Stirnimann, C., Lorber, B., Terrier, N., Ferrigno, R. & Sauter, C. (2013). *Cryst. Growth Des.* **13**, 3333–3340.

[bb130] Rossmann, M. G. (1979). *J. Appl. Cryst.* **12**, 225–238.

[bb131] Sauter, N. K. (2015). *J. Synchrotron Rad.* **22**, 239–248. 10.1107/S1600577514028203 PMC4344359 25723925

[bb134] Sauter, N. K., Hattne, J., Brewster, A. S., Echols, N., Zwart, P. H. & Adams, P. D. (2014). *Acta Cryst.* D**70**, 3299–3309. 10.1107/S1399004714024134 PMC4257623 25478847

[bb136] Sauter, N. K., Hattne, J., Grosse-Kunstleve, R. W. & Echols, N. (2013). *Acta Cryst.* D**69**, 1274–1282. 10.1107/S0907444913000863 PMC3689530 23793153

[bb138] Sauter, N. K., Kern, J., Yano, J. & Holton, J. M. (2020). *Acta Cryst.* D**76**, 176–192. 10.1107/S2059798320000418 PMC7008510 32038048

[bb140] Schlichting, I. (2017). *IUCrJ*, **4**, 516. 10.1107/S2052252517012167 PMC5619844 28989708

[bb142] Schreurs, A. M. M., Xian, X. & Kroon-Batenburg, L. M. J. (2010). *J. Appl. Cryst.* **43**, 70–82.

[bb144] Seijas-Macías, A. & Oliveira, A. (2012). *Discuss. Math. Probab. Stat.* **32**, 87–99.

[bb146] Shapiro, L., Fannon, A. M., Kwong, P. D., Thompson, A., Lehmann, M. S., Grübel, G., Legrand, J.-F., Als-Nielsen, J., Colman, D. R. & Hendrickson, W. A. (1995). *Nature*, **374**, 327–337. 10.1038/374327a0 7885471

[bb148] Sharma, A., Johansson, L., Dunevall, E., Wahlgren, W. Y., Neutze, R. & Katona, G. (2017). *Acta Cryst.* A**73**, 93–101. 10.1107/S2053273316018696 PMC5332129 28248658

[bb149] Sikorski, M., Feng, Y., Song, S., Zhu, D., Carini, G., Herrmann, S., Nishimura, K., Hart, P. & Robert, A. (2016). *J. Synchrotron Rad.* **23**, 1171–1179. 10.1107/S1600577516010869 27577772

[bb151] Spence, J. C. H. (2017). *IUCrJ*, **4**, 322–339. 10.1107/S2052252517005760 PMC5571796 28875020

[bb153] Stagno, J. R., Liu, Y., Bhandari, Y. R., Conrad, C. E., Panja, S., Swain, M., Fan, L., Nelson, G., Li, C., Wendel, D. R., White, T. A., Coe, J. D., Wiedorn, M. O., Knoska, J., Oberthuer, D., Tuckey, R. A., Yu, P., Dyba, M., Tarasov, S. G., Weierstall, U., Grant, T. D., Schwieters, C. D., Zhang, J., Ferré-D’Amaré, A. R., Fromme, P., Draper, D. E., Liang, M., Hunter, M. S., Boutet, S., Tan, K., Zuo, X., Ji, X., Barty, A., Zatsepin, N. A., Chapman, H. N., Spence, J. C. H., Woodson, S. A. & Wang, Y. (2017). *Nature*, **541**, 242–246. 10.1038/nature20599 PMC5502819 27841871

[bb156] Steller, I., Bolotovsky, R. & Rossmann, M. G. (1997). *J. Appl. Cryst.* **30**, 1036–1040.

[bb158] Terwilliger, T. C., Bunkóczi, G., Hung, L.-W., Zwart, P. H., Smith, J. L., Akey, D. L. & Adams, P. D. (2016). *Acta Cryst.* D**72**, 346–358. 10.1107/S2059798315019269 PMC4784666 26960122

[bb161] Thomaston, J. L., Woldeyes, R. A., Nakane, T., Yamashita, A., Tanaka, T., Koiwai, K., Brewster, A. S., Barad, B. A., Chen, Y., Lemmin, T., Uervirojnangkoorn, M., Arima, T., Kobayashi, J., Masuda, T., Suzuki, M., Sugahara, M., Sauter, N. K., Tanaka, R., Nureki, O., Tono, K., Joti, Y., Nango, E., Iwata, S., Yumoto, F., Fraser, J. S. & DeGrado, W. F. (2017). *Proc. Natl Acad. Sci. USA*, **114**, 13357–13362. 10.1073/pnas.1705624114 PMC5754760 28835537

[bb164] Tosha, T., Nomura, T., Nishida, T., Saeki, N., Okubayashi, K., Yamagiwa, R., Sugahara, M., Nakane, T., Yamashita, K., Hirata, K. *et al.* (2017). *Nat. Commun.* **8**, 1–9. 10.1038/s41467-017-01702-1 PMC5691058 29147002

[bb166] Uervirojnangkoorn, M., Zeldin, O. B., Lyubimov, A. Y., Hattne, J., Brewster, A. S., Sauter, N. K., Brunger, A. T. & Weis, W. I. (2015). *eLife*, **4**, e05421. 10.7554/eLife.05421 PMC4397907 25781634

[bb168] Waterman, D. G., Winter, G., Gildea, R. J., Parkhurst, J. M., Brewster, A. S., Sauter, N. K. & Evans, G. (2016). *Acta Cryst.* **72**, 558–575. 10.1107/S2059798316002187 PMC4822564 27050135

[bb170] White, T. A. (2014). *Philos. Trans. R. Soc. B*, **369**, 20130330. 10.1098/rstb.2013.0330 PMC4052866 24914157

[bb172] White, T. A., Kirian, R. A., Martin, A. V., Aquila, A., Nass, K., Barty, A. & Chapman, H. N. (2012). *J. Appl. Cryst.* **45**, 335–341.

[bb174] White, T. A., Mariani, V., Brehm, W., Yefanov, O., Barty, A., Beyerlein, K. R., Chervinskii, F., Galli, L., Gati, C., Nakane, T., Tolstikova, A., Yamashita, K., Yoon, C. H., Diederichs, K. & Chapman, H. N. (2016). *J. Appl. Cryst.* **49**, 680–689. 10.1107/S1600576716004751 PMC4815879 27047311

[bb176] Winter, G., Waterman, D. G., Parkhurst, J. M., Brewster, A. S., Gildea, R. J., Gerstel, M., Fuentes-Montero, L., Vollmar, M., Michels-Clark, T., Young, I. D., Sauter, N. K. & Evans, G. (2018). *Acta Cryst.* D**74**, 85–97. 10.1107/S2059798317017235 PMC5947772 29533234

[bb178] Yefanov, O., Mariani, V., Gati, C., White, T. A., Chapman, H. N. & Barty, A. (2015). *Opt. Express*, **23**, 28459–28470. 10.1364/OE.23.028459 PMC4646514 26561117

[bb180] Zhu, D., Cammarata, M., Feldkamp, J. M., Fritz, D. M., Hastings, J. B., Lee, S., Lemke, H. T., Robert, A., Turner, J. L. & Feng, Y. (2012). *Appl. Phys. Lett.* **101**, 034103.

