# Supplementary material for: Beyond integration: modeling every pixel to obtain better structure factors from stills
Source: IUCrJ. 2020 Oct 24;7(Pt 6):1151–67. doi: 10.1107/S2052252520013007 (PMC7642780; doi:10.1107/S2052252520013007)
Supplement: Supplementary file 1 [file m-07-01151-sup1.pdf]

IUCrJ

Volume 7 (2020)

Supporting information for article:

Beyond integration: modeling every pixel to obtain better structure factors from stills

Derek Mendez, Robert Bolotovsky, Asmit Bhowmick, Aaron S. Brewster, Jan Kern, Junko Yano, James M. Holton and Nicholas K. Sauter

Supplemental Information

Beyond integration: modeling every pixel to obtain better structure factors from stills

Derek Mendez[a], Robert Bolotovsky[a], Asmit Bhowmick[a], Aaron S. Brewster[a], Jan Kern[a], Junko Yano[a], James M. Holton[a,b,c], and Nicholas K. Sauter[a]

[a]Molecular Biophysics and Integrated Bioimaging Division (MBIB), Lawrence Berkeley National Lab, Berkeley, CA 94720 USA

[b]Stanford Synchrotron Radiation Lightsource, SLAC National Accelerator Laboratory, Menlo Park, CA 94025 USA

[c]Department of Biochemistry and Biophysics, UC San Francisco, San Francisco, CA 94158 USA

Contents

Supplemental Figures **2**

Supplemental Tables **9**

Supplemental Text **11**

S1 Mosaicity . . . 11

S2 Comparison with a profile fit approach . . . 11

S3 Detector geometry . . . 12

S4 Accounting for a detector point-spread function . . . 13

Supplemental Figures

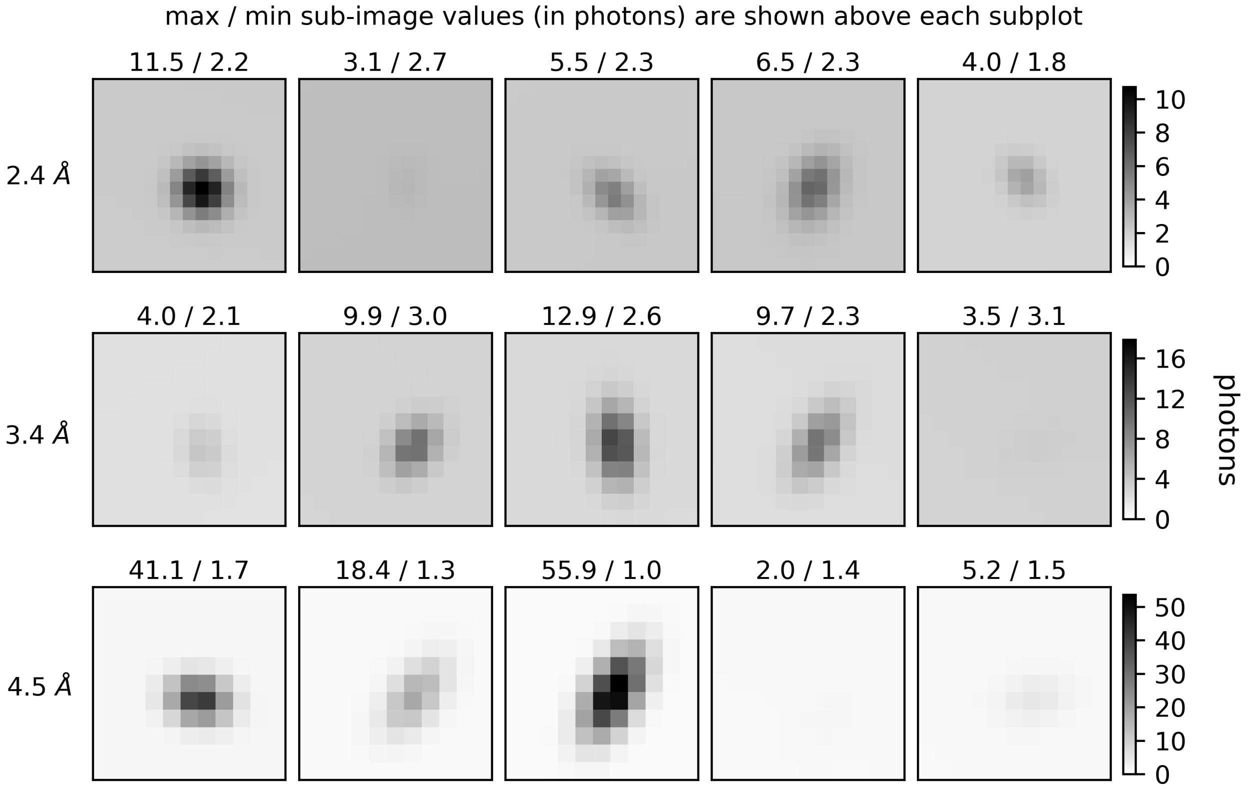

Figure S1: Synthetic data without noise. These images correspond to the main-text Figure 2, before adding random measurement noise. The maximum / minimum pixel values within each subplot image are shown for reference (photon units). The pixel values represent $I_{i,s,\mathrm{data}}+T_{i,s,\mathrm{data}}$ in the main text, *i.e.* the sum of expected Bragg and background scattering.

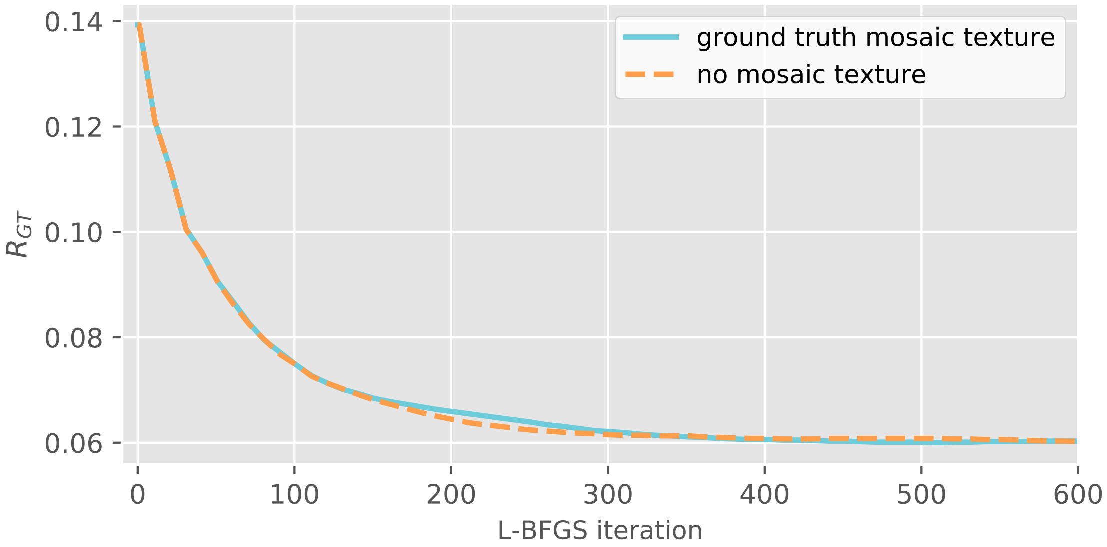

Figure S2: Structure factor refinement for different mosaicity models. The ground truth mosaic texture used to generate the data was applied during refinement, but it had little effect on the optimization. This is likely because the synthetic mosaic spread was relatively small (0.01°), and mosaic spread is a secondary effect that's dominated by mosaic domain size, especially at lower scattering angles.

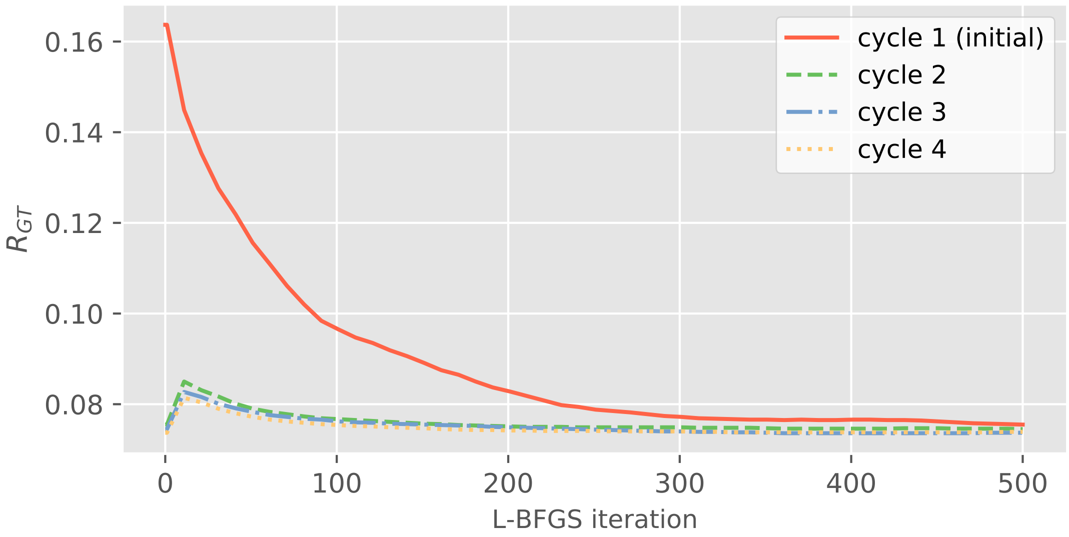

Figure S3: Repeating stage 1 and stage 2 refinements for 513 shots. After the initial stage 1 and stage 2 *diffBragg* refinements, we used the optimized structure factors and crystal models to conduct additional refinement cycles. The overall R_{GT} decreased from 0.076 to 0.075 upon completion of the second cycle, then down to 0.074 after a third cycle. After a fourth cycle, R_{GT} seemed to have converged at 0.074. Note, the shots used in this simple example were synthesized with a single mosaic domain and a single wavelength per shot, in contrast with the shots synthesized for the main paper, hence why the R-factors are different from those reported for 505 shots in the main text. The initial rise in R_{GT} for cycles 2, 3, and 4 occurred because the initial scale factor G_s for stage 2 of those cycles was set as the median value determined from cycle 1 / stage 1, before optimizing structure factors, and therefore was slightly inaccurate when used together with optimized structure factors obtained during cycle 1 / stage 2. In each case, the optimization corrected for this after about 10 iterations, then proceeded to minimize.

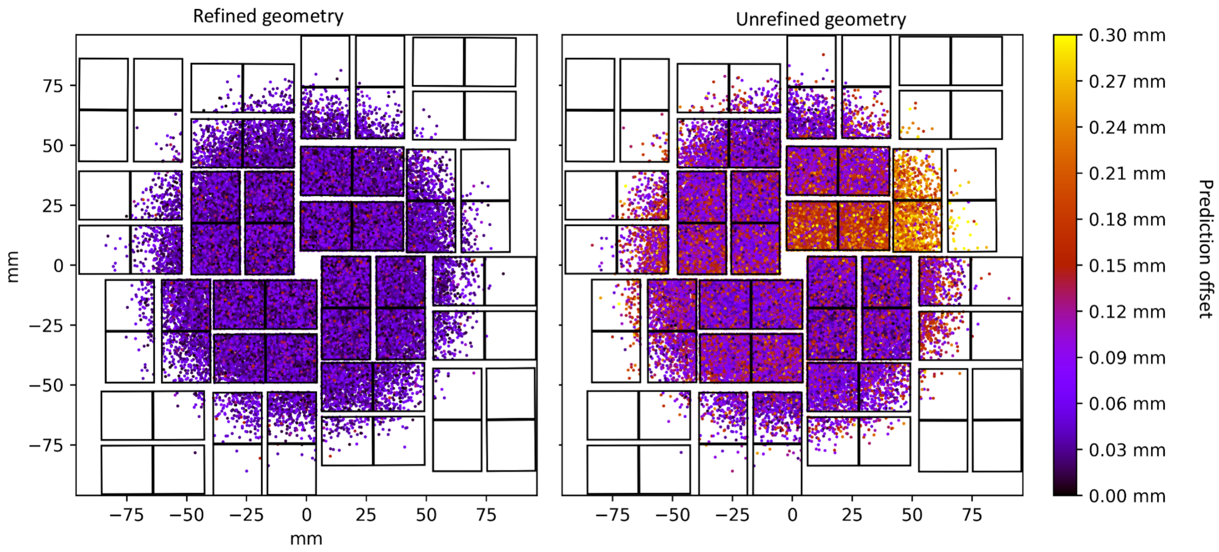

Figure S4: Refined and unrefined detector geometries. Spots represent positions of observed Bragg spots on the CSPAD which were successfully indexed, and the color of each spot represents the distance to the corresponding prediction. Anisotropy in such a plot is indicative of geometry inaccuracies. The unrefined geometry used here is representative of a typical starting geometry at LCLS. With the unrefined geometry, the per-panel prediction offset had a median value of 1.1 pixels, and a maximum value of 2.5 pixels. After refinement (Brewster *et al.* (2018)), these numbers reduced to 0.51 pixels and 0.69 pixels, respectively.

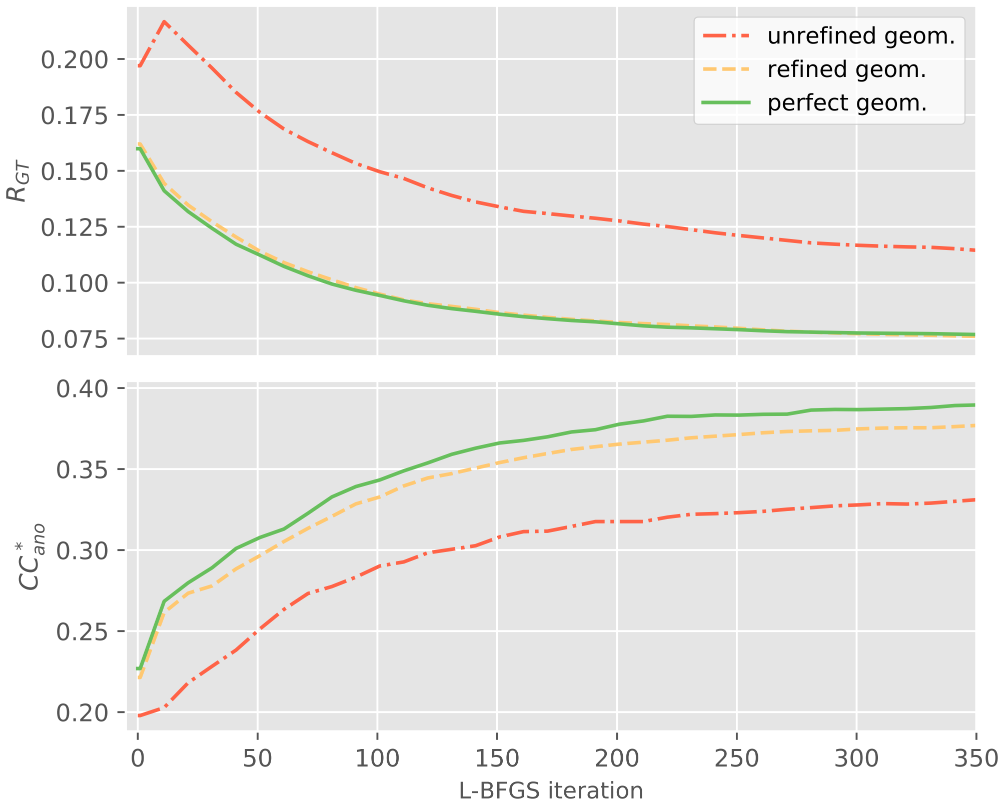

Figure S5: Structure factor refinement in the presence of “unrefined geometry”, “refined geometry” and “perfect geometry”. Each curve represents 459 shots which indexed successfully with all three geometries. Remarkably, refinement proceeds to converge even when subject to the highly erroneous “unrefined geometry”; the converged result is worse overall, given the panel position inaccuracies, but nevertheless a large improvement over integration-based merging (the integration-based merge results are the values of the curves at L-BFGS iteration 0). After indexing the 459 shots using the “unrefined geometry”, we ran the *CCTBX* script *cspad.cbf_metrology* (Brewster *et al.* (2018)) to obtain the “refined geometry”. We then ran stage 1 and stage 2 refinement using the “refined geometry” and achieved a result much closer to that obtained using the ground truth (perfect) geometry.

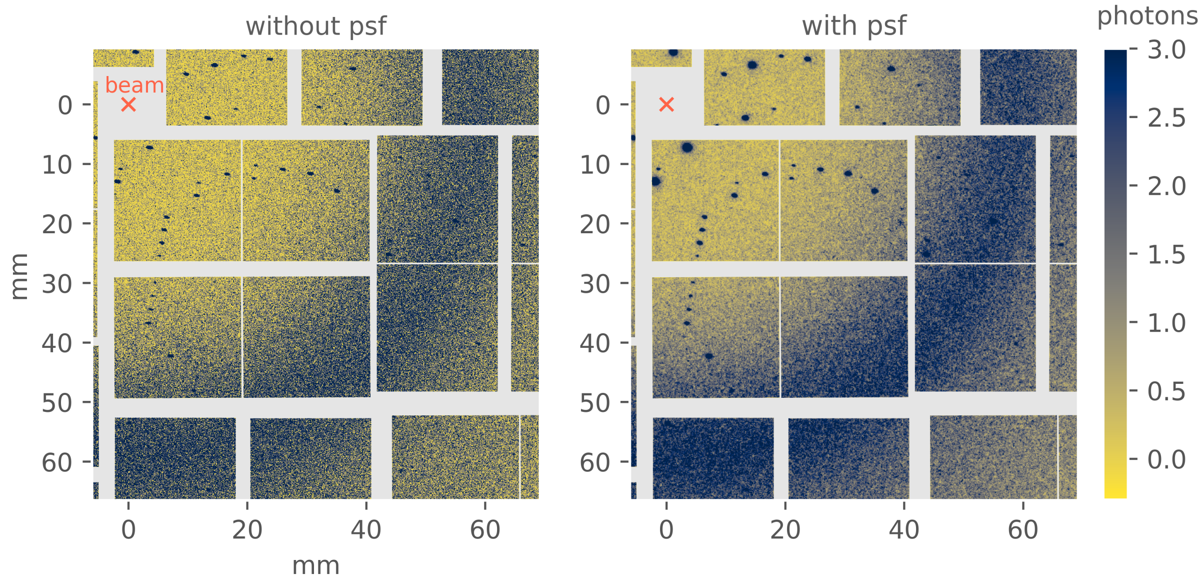

Figure S6: Application of a point-spread function to a synthetic diffraction pattern. The image is zoomed-in on a lower quadrant of the CSPAD (the forward beam is shown for reference). The point-spread kernel used here is typical of that observed on Rayonix cameras (Holton *et al.* (2012)), commonly used in SFX. The colorscale is the same for both images, and the upper limit of 3 photons was chosen to emphasize the point-spread effect.

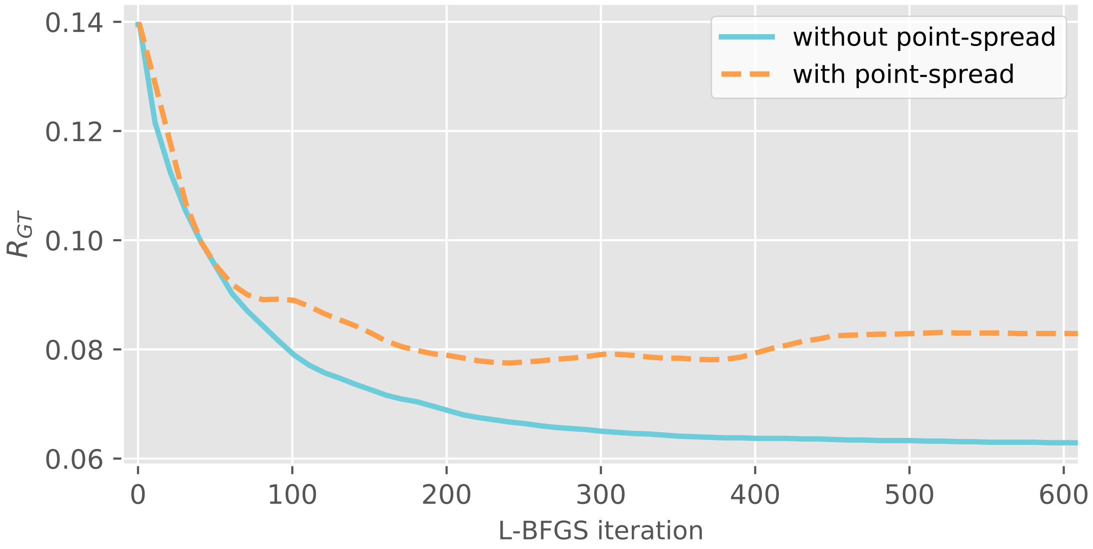

Figure S7: Refinement of structure factors in the presence of significant detector point-spread. Shots used here were synthesized according to the description in the main text, however we only used one mosaic domain and one photon energy per shot. We show here a stage 2 refinement using 495 synthetic shots, both with and without detector point-spread (see right and left panels of Figure S6, respectively). The same set of pixels was modeled in both cases such that the only difference between the refinements was the point-spread function. In spite of the point-spread function, structure factor refinement converged with improved accuracy, provided the point-spread kernel was applied to the model intensities and gradients (as in equations (S7) and (S8)) during optimization.

Supplemental Tables

	**Merge without post-refinement**			
Number of shots	R_{GT}(%)	CC^{*}_{ano} (%)	R_{split}(%)	CC 1/2 (%)
2023(1629)	10.9	49.1	9.4(74.0)	99.7(43.3)
6144(4982)	10.8	70.0	5.5(46.2)	99.7(73.1)
19953(15989)	10.2	85.7	3.2(25.6)	99.8(90.2)
	**Merge with post-refinement**			
Number of shots	R_{GT}(%)	CC^{*}_{ano} (%)	R_{split}(%)	CC 1/2 (%)
2023(1489)	7.8	49.5	8.8(82.2)	99.8(28.9)
6144(4525)	6.7	71.2	4.9(47.8)	99.9(70.3)
19953(14643)	7.1	85.8	2.6(27.0)	100(90.4)

Table S1: Integration-based *CCTBX* merging, with and without post-refinement (values in parenthesis are at the high resolution bin). *CCTBX* merging with the command line script *cxi.merge* (or *cctbx.xfel.merge*) uses a per-image resolution cutoff, hence why the number of shots contributing to the high resolution bin is lower than the total number of shots used. One can merge data with the option *post_refinement.enable=True*, however doing so requires a reference model, either in the form of a PDB file or a structure factor MTZ file. In this case, we are assuming we do not know the PDB model, however we can use the structure factor table obtained without post-refinement as the reference model for a merge with post refinement (see also Brewster *et al.* (2019)). Here, overall statistics improve with post-refinement, however high-resolution statistics worsen, due to additional shot rejection imposed by the post-refinement algorithm (note the consistently fewer number of shots used in the high resolution bins for the post-refinement merges). Crucially, CC^{*}_{ano}, which is the preferred metric for predicting the ability to phase a dataset (Terwilliger *et al.* (2016)), minimally improves with post-refinement. Note, the "no post-refinement" merge statistics shown here differ slightly from those shown in main-text Table 5. Though negligible, this results from using a slightly different set of *cxi.merge* arguments.

	**I**: $\langle I_{\mathbf{h}}\rangle$		**II**: $\langle I_{\mathbf{h}}/P_{\mathbf{h}}\rangle$		**III**: diffBragg stage 2	
number of shots	R_{GT}	CC^{*}_{ano}	R_{GT}	CC^{*}_{ano}	R_{GT}	CC^{*}_{ano}
505	0.155	0.230	0.165	0.298	0.059	0.479
2023	0.136	0.458	0.141	0.570	0.049	0.790
6144	0.131	0.657	0.136	0.753	0.049	0.904

Table S2: Comparing alternate integration merging methods with diffBragg structure factor refinement (stage 2). Note, these merge methods are outside of the *CCTBX* scope, hence why the "column **I**" results differ from those reported for the integration method in main-text Table 5.

metric	perfect geometry	unrefined geom.	refined geom.
misorientation from *dials.stills_process* (deg.)	0.038 ± 0.028	0.079 ± 0.03	0.039 ± 0.026
... after *diffBragg* stage 1	0.0035 ± 0.0024	0.079 ± 0.037	0.0074 ± 0.003
unit cell a from *dials.stills_process* (Å)	79.095 ± 0.010	79.32 ± 0.050	79.099 ± 0.011
... after *diffBragg* stage 1	79.097 ± 0.0048	79.29 ± 0.073	79.11 ± 0.0063
unit cell c from *dials.stills_process* (Å)	38.42 ± 0.058	38.53 ± 0.081	38.42 ± 0.056
... after *diffBragg* stage 1	38.40 ± 0.0048	38.49 ± 0.096	38.41 ± 0.0075
R_{GT} from *cctbx.xfel.merge*	0.16	0.21	0.16
... after *diffBragg* stage 2	0.075	0.11	0.074
CC^*_{ano} *cctbx.xfel.merge*	0.23	0.20	0.22
... after *diffBragg* stage 2	0.39	0.33	0.38

Table S3: Data quality metrics, and how they are influenced by geometry errors. The ground truth unit cell is $a = 79.1\,$Å, $c = 38.4\,$Å. The merge results shown are for 459 shots.

Supplemental Text

S1 Mosaicity

The synthetic data described in the manuscript was computed using a finite mosaic texture, with an effective mosaic spread of 0.01 deg. When we performed *diffBragg* refinement we made the decision to exclude mosaic spread from the model, on the basis that the mosaic domain size appeared to be a more dominant effect. This is illustrated in a general sense by Figure 7 of Sauter *et al.* (2014). To further justify the decision, we performed a "stage 2 *diffBragg* refinement" on a limited number of shots using the ground truth mosaic texture for each crystal and found that it did not improve the structure factor optimization (Figure S2). Modeling mosaic spread can be computationally costly, so in certain circumstances when the mosaic spread is seemingly small, it is much more efficient to leave it out of the model. One will note that the ground truth mosaic domain size parameter m was 10 for the synthetic data, indicating that each mosaic block consisted of 10 unit cells along each crystal axis. The optimized value for m however was slightly less than 10 (main-text Figure 3). We suspect this slight reduction in mosaic domain size occurred in order to account for a lack of mosaic spread in the model (smaller domain sizes result in larger spot profiles).

S2 Comparison with a profile fit approach

Here we explore how profile fitting using models resulting from "stage 1 *diffBragg* refinement" can enhance structure factor estimation in the conventional integration approach. With integration-based methods, structure factor estimates are obtained by summing up regions of pixels near predicted Bragg reflections. We model a summed spot integration as

$$I_{\mathbf{h}} = \sum_{i \in \text{spot}} I_{i,s} M_{i,s} \tag{S1}$$

where

$$I_{i,s} = J_{s,\text{all}} G_s r_e^2 |F_{\mathbf{h}}|^2 m_s^6 \exp\left(-C m_s^2 \Delta\mathbf{h}_{i,s} \cdot \Delta\mathbf{h}_{i,s}\right) \kappa_i \Delta\Omega_i \tag{S2}$$

is equation (15) of the main text and $M_{i,s}$ is the integration mask, i.e. it takes on values of $1, 0$ depending on whether the pixel is in the foreground, background, respectively. This same expression can be used to determine a per-spot correction factor $P_{\mathbf{h}}$. For each pixel, we computed

$$P_{i,s} = J_{s,\text{all}} G_s r_e^2 m_s^6 \exp\left(-C m_s^2 \Delta\mathbf{h}_{i,s} \cdot \Delta\mathbf{h}_{i,s}\right) \kappa_i \Delta\Omega_i \tag{S3}$$

such that the correction term is

$$P_{\mathbf{h}} = \sum_{i \in \text{spot}} P_{i,s} M_{i,s} \tag{S4}$$

After stage 1 *diffBragg* refinement, we used the optimized per-shot parameters $G_s, m_s, \mathbf{B}_s, \mathbf{U}_s$ (scale factors, mosaic parameters, unit cell matrices, and orientation matrices) to form integration masks

$$M_{i,s} = \begin{cases} 1 & \text{if } I_{i,s} > \chi \\ 0 & \text{otherwise} \end{cases} \tag{S5}$$

and to compute $I_{\mathbf{h}}$ and $P_{\mathbf{h}}$ for every indexed spot that entered the *diffBragg* refinement. The threshold was chosen to be $\chi = 0.01$ (χ is in units of photons). We then directly compared three methods for estimating structure factors:

I : integration averaging over equivalent reflections $|F_{\mathbf{h}}|^2 = \langle I_{\mathbf{h}} \rangle_{\text{equivalents}}$

II : integration averaging over equivalent reflections with profile correction $|F_{\mathbf{h}}|^2 = \langle I_{\mathbf{h}}/P_{\mathbf{h}} \rangle_{\text{equivalents}}$

III : *diffBragg* stage 2 optimization

The results for R_{GT} and CC^*_{ano} are shown in Table S2 for the various merges. Note, R_{GT} is consistently worse for method II, but CC^*_{ano} shows significant improvement, and is generally regarded as a more rigorous indicator of structure factor accuracy. Also, the results shown here for method I are slightly different than those shown in the main text for integration-based merging, as the main text integration-based merging was done using the command line program *cxi.merge*. In particular, stage 1 refinement is dependent on structure factor estimates, and initial errors in structure factor estimates will lead to errors in the post- stage 1 profile estimates $P_{\mathbf{h}}$. Indeed, in order to achieve improved accuracy with method II, we had to first filter $P_{\mathbf{h}}$ outliers amongst equivalent reflections using a median absolute deviation threshold (Iglewicz and Hoaglin (1993)). Without filtering for outliers, method II performed consistently worse than method I. This highlights the utility of stage 2 *diffBragg* refinement: rather than using a single number (summed pixels) to represent each Bragg spot's contribution to $F_{\mathbf{h}}$, it uses all pixels in the neighborhood of the corresponding Bragg spot, each contributing differently to the total data likelihood depending on the probability of observation. Further, *diffBragg* stage 2 allows one to obtain more accurate structure factor estimates by further refinement of the stage 1 model parameters *simultaneously* with the structure factor amplitudes.

S3 Detector geometry

The majority of XFEL diffraction cameras are made up of multiple pixel array detectors (PADs), and it is generally recognized that panel position inaccuracies plague XFEL data analysis. While programs exist that

use diffraction patterns to optimize the detector geometry (Yefanov *et al.* (2015); Brewster *et al.* (2018)), they are not perfect, and inaccuracies in panel positions are to be expected when dealing with real data. In order to test the stability of *diffBragg* refinement in the presence of unrefined geometry, we used a detector geometry model with very large, yet realistic errors (Figure S4) in order to process the synthetic data (referred to as "unrefined geometry"). We also optimized the "unrefined geometry" using the CCTBX command line program *cspad.cbf_metrology* (Brewster *et al.* (2018)), obtaining a geometry which we refer to as "refined geometry". Before optimization, the "unrefined geometry" had positional errors on the order of 1.1 pixels, up to 2.5 pixels on the panels with the largest errors. The "refined geometry" had errors on the order of 0.51 pixels, up to 0.69 pixels. Errors in geometry lower the quality of the analysis at every step of the pipeline (Table S3). Notably, "stage 2 *diffBragg* refinement" is stable in spite of the panel inaccuracies, even when no geometry optimization is performed on the "unrefined geometry" (Figure S5). The effects of geometry on integration-based merge quality are also illustrated in Figure 2 of Hattne *et al.* (2014).

The geometry we used to synthesize the data for the manuscript is referred to as "perfect geometry" or "ground truth geometry", and was taken directly from an experimental dataset (LCLS proposal number LD91) *after* optimizing panel orientations according to Brewster *et al.* (2018). The "unrefined geometry" used here is simply the original experimental geometry that was optimized against the real LD91 data to form the geometry we are calling "perfect geometry". Therefore, the degree of panel error present in "unrefined geometry" is typical of what one can expect at an XFEL beamline. Finally, the "refined geometry" was obtained by re-refining the "unrefined geometry" against the synthetic data (Figure S4).

S4 Accounting for a detector point-spread function

Though we developed *diffBragg* to work for newer generation pixel array detectors with minimal point spread, we demonstrate here that *diffBragg* can indeed be used to analyze data that includes a significant point-spread function. Following Holton *et al.* (2012), we applied a point-spread function to the synthesized diffraction patterns,

$$X_{i,s,\mathrm{psf}} = G * X_{i,s} \tag{S6}$$

where the $*$ is an image convolution operator, and G is a two-dimensional kernel function defined in the detector plane, and modeled here as a sum of two dimensional Gaussian terms (Holton *et al.* (2012)). Recall that the pixel index i is really a triple index $(panel, fast, slow)$ that indexes a multi-panel CSPAD camera (see main-text, section 2.1.4), however the kernel G used here is independent of the location of pixel i. Figure S6 shows the effect of the point-spread function when applied to the synthetic data. Point-spread modulates the intensity, so it should influence *diffBragg* refinement. Provided we estimate or measure the point-spread kernel G as in

Holton *et al.* (2012), we can account for it in *diffBragg* by applying it to the model (main-text, equation (15))

$$n_{i,s}(\Theta) \to G * n_{i,s}(\Theta) \tag{S7}$$

and the corresponding gradient terms (main-text, equation (23))

$$\frac{\partial n_{i,s}(\Theta)}{\partial \theta} \to G * \frac{\partial n_{i,s}(\Theta)}{\partial \theta} \tag{S8}$$

Figure S7 shows "*diffBragg* stage-2 refinement" with and without finite point-spread. Even if point-spread is present in the synthetic data, refinement proceeds, however the converged R-factor is 2% higher than it would be without point-spread.

References

Brewster, A. S., Bhowmick, A., Bolotovsky, R., Mendez, D., Zwart, P. H. and Sauter, N. K. (2019). *Acta Crystallographica Section D: Structural Biology*, **75**(11), 959–968.

Brewster, A. S., Waterman, D. G., Parkhurst, J. M., Gildea, R. J., Young, I. D., O'Riordan, L. J., Yano, J., Winter, G., Evans, G. and Sauter, N. K. (2018). *Acta Crystallographica Section D: Structural Biology*, **74**(9), 877–894.

Hattne, J., Echols, N., Tran, R., Kern, J., Gildea, R. J., Brewster, A. S., Alonso-Mori, R., Glöckner, C., Hellmich, J., Laksmono, H. *et al.* (2014). *Nature methods*, **11**(5), 545.

Holton, J. M., Nielsen, C. and Frankel, K. A. (2012). *Journal of synchrotron radiation*, **19**(6), 1006–1011.

Iglewicz, B. and Hoaglin, D. C. (1993). *How to detect and handle outliers*, vol. 16. Asq Press.

Sauter, N. K., Hattne, J., Brewster, A. S., Echols, N., Zwart, P. H. and Adams, P. D. (2014). *Acta Crystallographica Section D: Biological Crystallography*, **70**(12), 3299–3309.

Terwilliger, T. C., Bunkóczi, G., Hung, L.-W., Zwart, P. H., Smith, J. L., Akey, D. L. and Adams, P. D. (2016). *Acta Crystallographica Section D: Structural Biology*, **72**(3), 346–358.

Yefanov, O., Mariani, V., Gati, C., White, T. A., Chapman, H. N. and Barty, A. (2015). *Optics express*, **23**(22), 28459–28470.
